# 2,7-Diaminobenzopyrylium Dyes Are Live-Cell Mitochondrial Stains

**DOI:** 10.1021/acsbiomedchemau.1c00068

**Published:** 2022-02-28

**Authors:** Sambashiva Banala, Ariana N. Tkachuk, Ronak Patel, Pratik Kumar, Timothy A. Brown, Luke D. Lavis

**Affiliations:** Janelia Research Campus, Howard Hughes Medical Institute, 19700 Helix Drive, Ashburn, Virginia 20147, United States

**Keywords:** fluorescent probes, cellular imaging, chemical biology, organic chemistry, mitochondria, coumarin, benzopyrylium

## Abstract

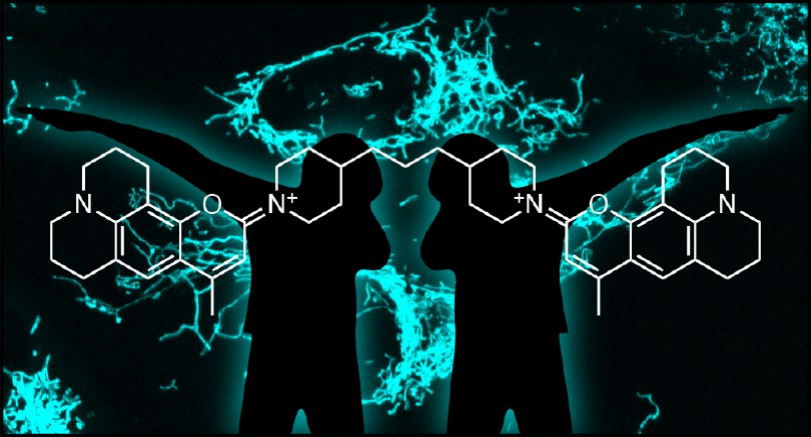

Small-molecule fluorescent stains enable the imaging of cellular structures without the need for genetic manipulation. Here, we introduce 2,7-diaminobenzopyrylium (DAB) dyes as live-cell mitochondrial stains excited with violet light. This amalgam of the coumarin and rhodamine fluorophore structures yields dyes with high photostability and tunable spectral properties.

## Introduction

Fluorescence microscopy is an essential tool to interrogate biological structure. A key element in any imaging experiment is the labeling strategy used to localize a fluorophore to the cellular component of interest.^1−5^ In addition to measuring the position and movement of specific biomolecules, cellular imaging experiments often involve the visualization of different organelles to quantify their dynamics^6^ or provide useful subcellular reference marks.^7−9^ Fusing a fluorescent protein to a targeting motif can allow labeling of cellular organelles but requires expression of an exogenous molecule. Fluorescent reagents with affinities for organelle-specific biomolecules can allow imaging without genetic manipulation but typically involve preparation of a small-molecule fluorophore conjugated to an antibody or drug. An alternative labeling strategy is the use of fluorescent stains that accumulate in specific organelles due to the different chemical environments found in these distinct subcellular regions. Examples include tertiary amine-containing dyes accumulating in acidic lysosomes^10^ or hydrophobic fluorophores partitioning into lipid droplets.^11^

A widely used fluorescent staining strategy involves mitochondria, whose double membrane structure reflects their role as the powerhouse of the cell. The proteins that comprise the electron transport chain reside in the inner mitochondrial membrane that separates the matrix from the intermembrane space. Their activity drives protons across the inner membrane, resulting in a large voltage difference between these two compartments. This unique membrane potential drives the accumulation of lipophilic cations into the matrix or inner membrane. This was first observed with Rhodamine 123 (**1**),^12^ where esterification of the standard *ortho*-carboxyl group found in rhodamines endows the molecule with a fixed cationic charge. The strategy was expanded to tetramethylrhodamine methyl ester (TMRM, **2**), yielding a red-shifted mitochondrial stain.^13^ This general idea was refined with the development of MitoTracker Orange (**3**), in which the carboxyl ester functionality found in rhodamines **1** and **2** is discarded entirely.^7^ Compound **3** also incorporates a chloromethyl moiety to allow formation of a glutathione adduct, thereby trapping the fluorophore inside the cell.^14^

The majority of fluorescent mitochondrial stains are based on rhodamine (e.g., **1**–**3**) and cyanine structures.^7^ These dyes exhibit relatively long absorption maxima (λ_abs_) and fall into the standard blue (488 nm), green-yellow (560 nm), and red (640 nm) excitation windows used in fluorescence imaging. Mitochondrial stains excited with violet light (405 nm) have received less attention since there is no general cationic dye scaffold with an excitation maximum in this wavelength range. We sought, and now report, a new class of mitochondrial stains based on 2,7-diaminobenzopyrylium (DAB) dyes.

## Results and Discussion

**Figure 1:**
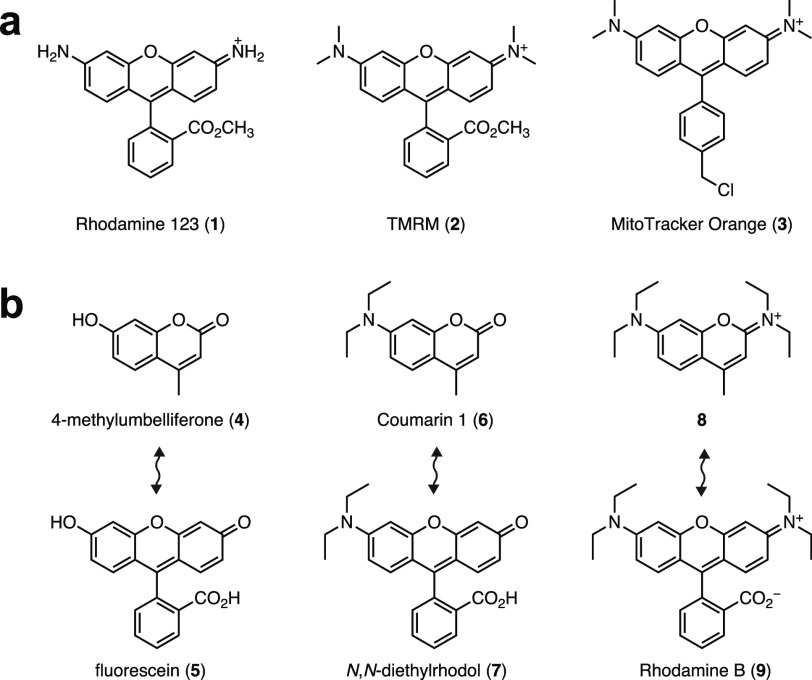
2,7-Diaminobenzopyrylium (DAB) dyes as potential mitochondrial stains. (a) Chemical structures of mitochondrial stains **1**–**3**. (b) Pairs of structurally consonant dyes: 4-methylumbelliferone (**4**) and fluorescein (**5**); Coumarin 1 (**6**) and *N*,*N*-diethylrhodol (**7**); 2,7-diaminobenzopyrylium **8** and Rhodamine B (**9**).

To create a violet-excited mitochondrial stain, we first considered coumarin dyes, which remain the most utilized fluorophores excited by ultraviolet (UV) and violet light. The simplest fluorescent coumarins are 7-hydroxy derivatives such as 4-methylumbelliferone (**4**, Figure 1b). We noted that the classic fluorophore fluorescein (**5**) is effectively the phenylogous derivative of **4**. Similarly, 7-aminocoumarins such as Coumarin 1 (**6**) are structurally consonant with rhodols like **7**. Inspired by the structural relationship between compounds **4**/**5** and **6**/**7**, we considered 2,7-diaminobenzopyrylium (DAB) structures exemplified by the tetraethyl derivative **8**; this is the “coumarin-sized” analog of Rhodamine B (**9**). Although iminocoumarins have received some attention as dyes^15^ and indicators,^16^ the cationic 2,7-diaminobenzopyrylium fluorophore scaffold is essentially unexplored, with a lone report in the Soviet chemistry literature from 1989.^17^

**Scheme 1:**
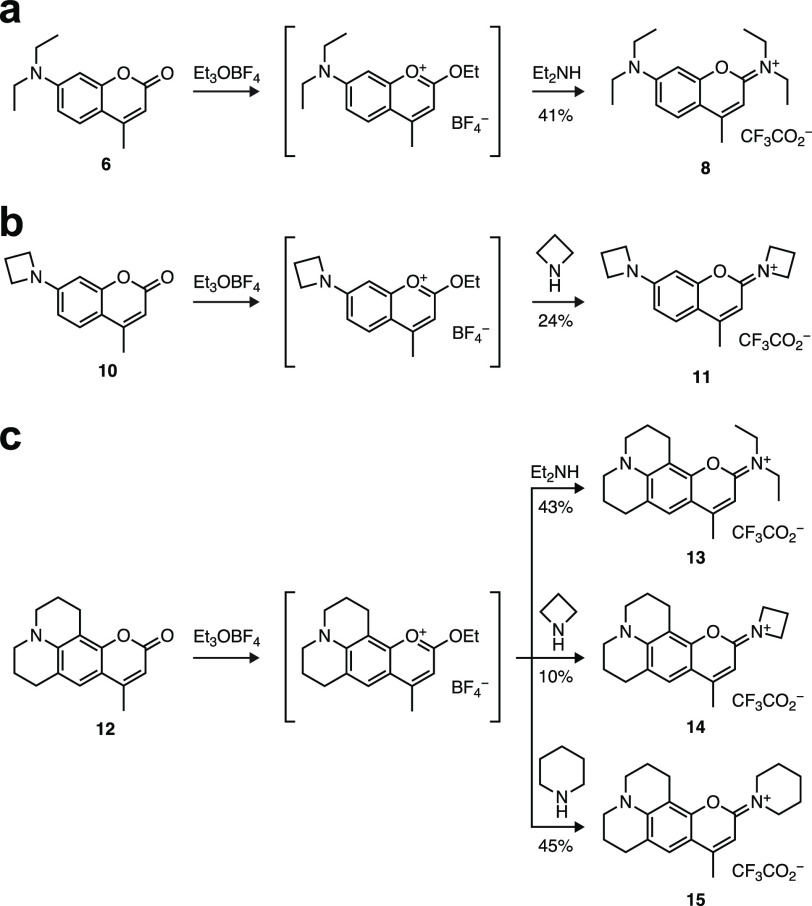
Synthesis of dyes **8** (a), **11** (b), and **13**–**15** (c)

We began our investigation by synthesizing the known tetraethyl DAB dye **8** starting from Coumarin 1 (**6**, Scheme 1a).^17^ Treatment with Et_3_OBF_4_ generates a 2-ethoxychromenylium intermediate that reacts with diethylamine to give **8**, which we obtained in 41% yield. Based on the success of this simple synthetic protocol, we prepared additional DAB dyes. We transformed the bright azetidinylcoumarin^18^**10** into the diazetidinyl dye **11** (Scheme 1b). We also explored dyes derived from Coumarin 102 (**12**); reaction with diethylamine, azetidine, or piperidine yielded compounds **13**–**15** (Scheme 1c).

**Figure 2:**
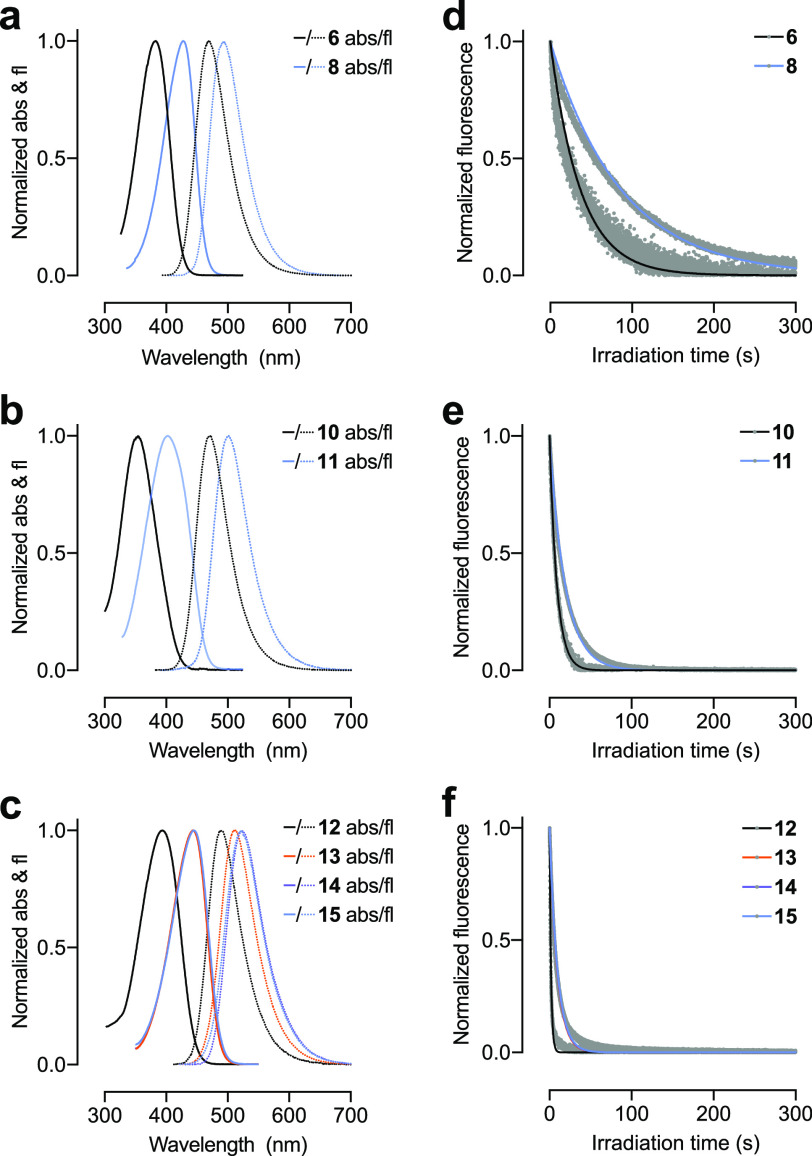
Spectra and photobleaching of representative DAB dyes. (a–c) Normalized absorption (abs) and fluorescence emission (fl) of (a) **6** and **8**, (b) **10** and **11**, and (c) **12**–**15**. (d–f) Plot of normalized fluorescence vs irradiation time of (d) **6** and **8**, (e) **10** and **11**, and (f) **12**–**15** with monoexponential fit.

We then evaluated the spectral properties of the DAB dyes **8**, **11**, and **13**–**15** in phosphate-buffered saline (PBS; Table 1, Figure 2a–c) comparing them to the parent coumarin fluorophores **6**, **10**, and **12**. In general, the transformation of the carbonyl group into an iminium moiety elicits a bathochromic shift of ∼50 nm in λ_abs_ and a shift of 25–35 nm in fluorescence emission maxima (λ_em_). Thus, all the DAB dyes exhibit λ_abs_ > 400 nm, absorbing in the violet-blue region of the visible spectrum. The reduced shift in λ_em_ results in smaller Stokes shifts for the DAB fluorophores relative to the coumarin starting materials. Despite this decrease, the Stokes shifts of the DAB dyes (65–100 nm) remain substantially larger than those of fluoresceins or rhodamines (∼25 nm). Finally, for each matched pair, the DAB congeners show substantially higher photobleaching time constants (*t*_b_) and average number of photons emitted before photobleaching (*N*_p_) relative to the corresponding coumarin dye.

**Table 1:**
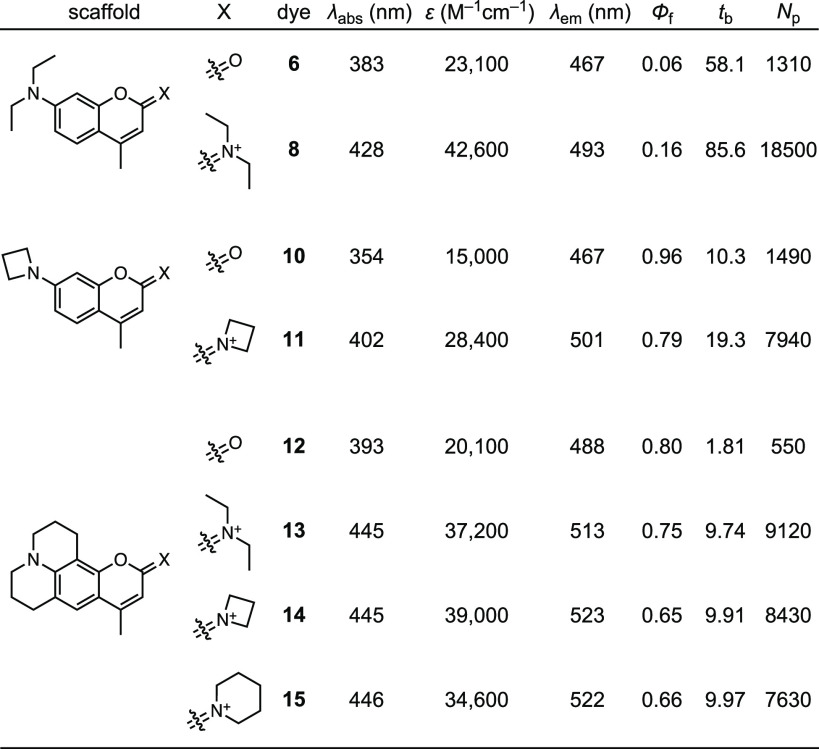
Spectral Properties of DAB Dyes^a^

a All values measured in PBS, pH 7.4, solution.

Comparison of specific dye pairs reveal more nuanced differences. The transformation of Coumarin 1 (**6**) to DAB dye **8** yields a shift of 45 nm in λ_abs_ and 26 nm in λ_em_, resulting in a Stokes shift of 65 nm (Table 1, Figure 2a). Conversion of coumarin **10** into diazetidinyl DAB derivative **11** results in a bathochromic shift of 48 nm in λ_abs_ and a difference of 34 nm in λ_em_ (Table 1, Figure 2b). This larger shift in emission maximum combined with the properties of the parent coumarin **10** gives a Stokes shift of 99 nm for **11**. For DAB dyes **13**–**15** derived from Coumarin 102 (**12**), the λ_abs_ does not depend on the different secondary amine auxochromes (Table 1, Figure 2c). The fluorescence emission does vary with structure; the acyclic *N*,*N*-diethylamino derivative **13** exhibits λ_em_ = 513 nm, which is 10 nm shorter than **14** and **15**, resulting in a smaller Stokes shift of 68 nm.

Across the series, we found that the absorptivity of the DAB dyes is substantially higher than the parent coumarin fluorophores, with 1.5–2-fold increases in extinction coefficient at λ_abs_ (ε, Table 1). The transformation of the coumarin oxygen into an iminium moiety elicits variable effects on fluorescence quantum yield (Φ_f_). For the relatively dim Coumarin 1 (**6**; Φ_f_ = 0.06) conversion to DAB dye **8** increases quantum yield (Φ_f_ = 0.16). In contrast, diazetidinyl dye **11** shows a modestly lower quantum yield (Φ_f_ = 0.79) compared to the bright azetidinyl coumarin **10** (Φ_f_ = 0.96). The DAB dyes **13**–**15** exhibit lower Φ_f_ values compared to **12**. These modest decreases in Φ_f_ for compounds **11** and **13**–**15** are balanced by the larger ε values, resulting in higher molecular brightness (ε × Φ_f_) for the DAB dyes relative to the corresponding coumarins.

As mentioned above, the DAB dyes exhibit increased photostability compared to their coumarin congeners. Dyes **8** and **11** gave 1.5–2-fold longer *t*_b_ values compared to coumarins **6** and **10** (Table 1, Figure 2d,e). For compounds **13**–**15**, the photostability improvement is greater, with these DAB compounds showing consistent *t*_b_ values that are 5-fold higher than the parent coumarin **12** (Table 1, Figure 2d). Since photobleaching reactions stem from excited states, it is difficult to compare photobleaching time constants between dyes with different fluorescence quantum yields and lifetimes; this is reflected in the different bleaching rates observed across the various dye types (Table 1, Figure 2d–f). We therefore calculated the average total photons (*N*_p_) emitted by each dye.^19^ This parameter highlights the increased photostability of the DAB fluorophores and revealed remarkably consistent photostability for compounds **11** and **13**–**15** with an average of ∼8300 photons/dye (Table 1).

**Figure 3:**
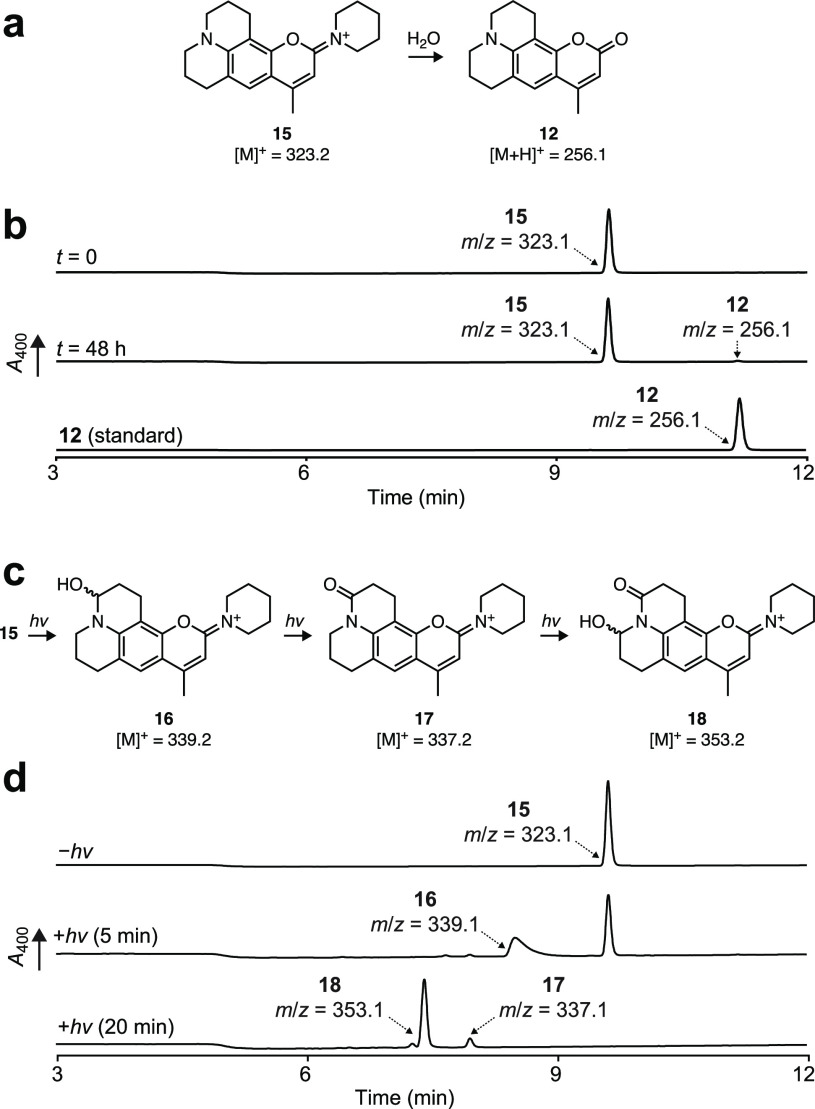
Stability of DAB dye **15**. (a) Spontaneous hydrolysis of **15** to form coumarin **12**. (b) LC–MS chromatograms of **15** at *t* = 0 (top), **15** at *t* = 48 h (middle), and standard **12** (bottom) in PBS, pH 7.4. (c) Photochemistry of **15** to form oxidized adducts **16**–**18**. (d) LC–MS chromatograms in PBS, pH 7.4, of **15** in the absence of light (top), **15** after 5 min illumination with 405 nm light (middle), and **15** after 20 min illumination with 405 nm light (bottom).

We then evaluated the stability of the iminium linkage in aqueous solution under “dark” conditions and under illumination with violet (405 nm) light. Monitoring with tandem liquid chromatography–mass spectrometry (LC–MS) revealed that all the DAB dyes (**8**, **11**, and **13**–**15**) show excellent stability with minimal iminium hydrolysis after 48 h at pH 7.4 in the absence of light (Figure 3a,b, Figure S1). We also evaluated the stability of dye **15** in different buffer conditions, observing modestly higher rates of hydrolysis at elevated pH or in cell culture media containing serum (Figure S2). We then undertook a comprehensive photochemistry study of **15** using 405 nm illumination and analysis by LC–MS (Figure 3c,d, Figure S3) The photochemical reactions of **15** are similar to those observed with parent coumarin **12** where oxidation appears to be centered on the julolidine ring system;^20,21^ we did not detect any photochemically driven oxidation on the piperidine ring. Overall, these data show that the DAB dyes exhibit reasonable chemical stability and the iminium motif is not susceptible to photochemical degradation.

**Figure 4:**
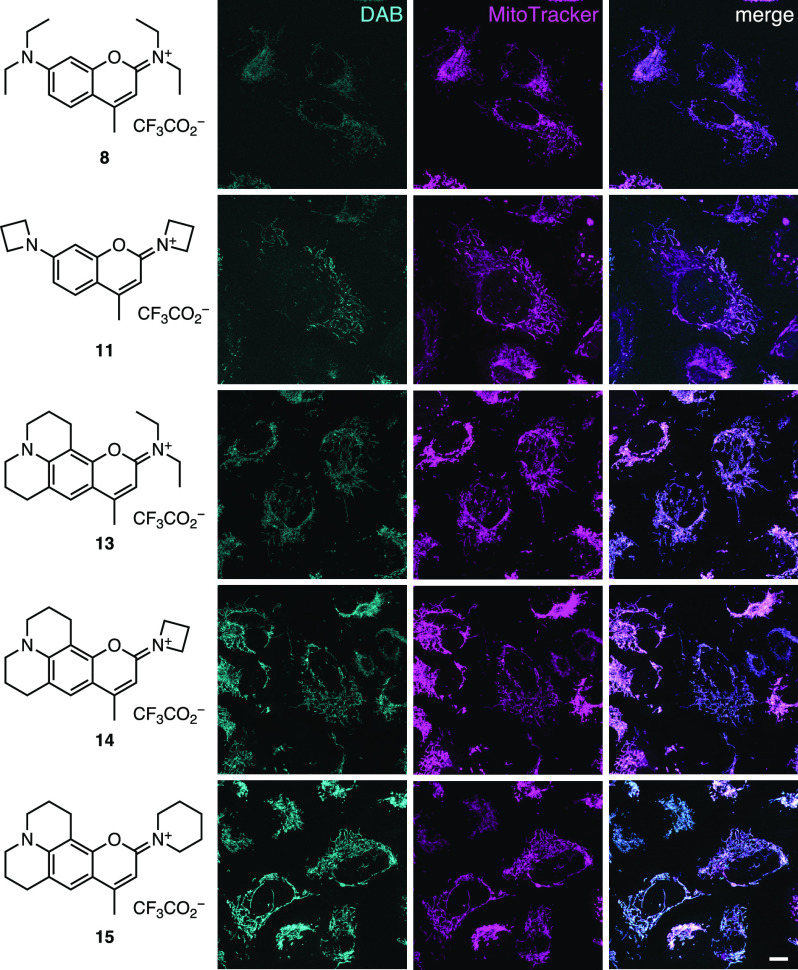
DAB dyes stain mitochondria. Chemical structures of DAB dyes **8**, **11**, and **13**–**15** and corresponding fluorescence images of U2OS cells co-stained with the DAB dye (200 nM) and MitoTracker Deep Red (100 nM). Scale bar, 10 μm.

Given the propensity of cationic rhodamines to accumulate in the mitochondria (Figure 1a), we investigated the similarly charged DAB dyes as live-cell mitochondrial stains. We incubated live U2OS cells with dyes **8**, **11**, and **13**–**15** at 200 nM and co-stained the mitochondria either using MitoTracker Deep Red (Figure 4) or by transient transfection of HaloTag–TOMM20 and staining with the far-red fluorogenic label Janelia Fluor 635-HaloTag ligand^22^ (Figure S4). In both experiments, we observed similar staining patterns between the DAB dyes and the established far-red stain or genetically encoded label, confirming our hypothesis that this positively charged fluorophore scaffold would accumulate in mitochondria. Imaging using the same settings revealed that the julolidine-containing derivatives **13**–**15** showed brighter staining, perhaps due to increased lipophilicity of the compact cationic structure.

**Figure 5:**
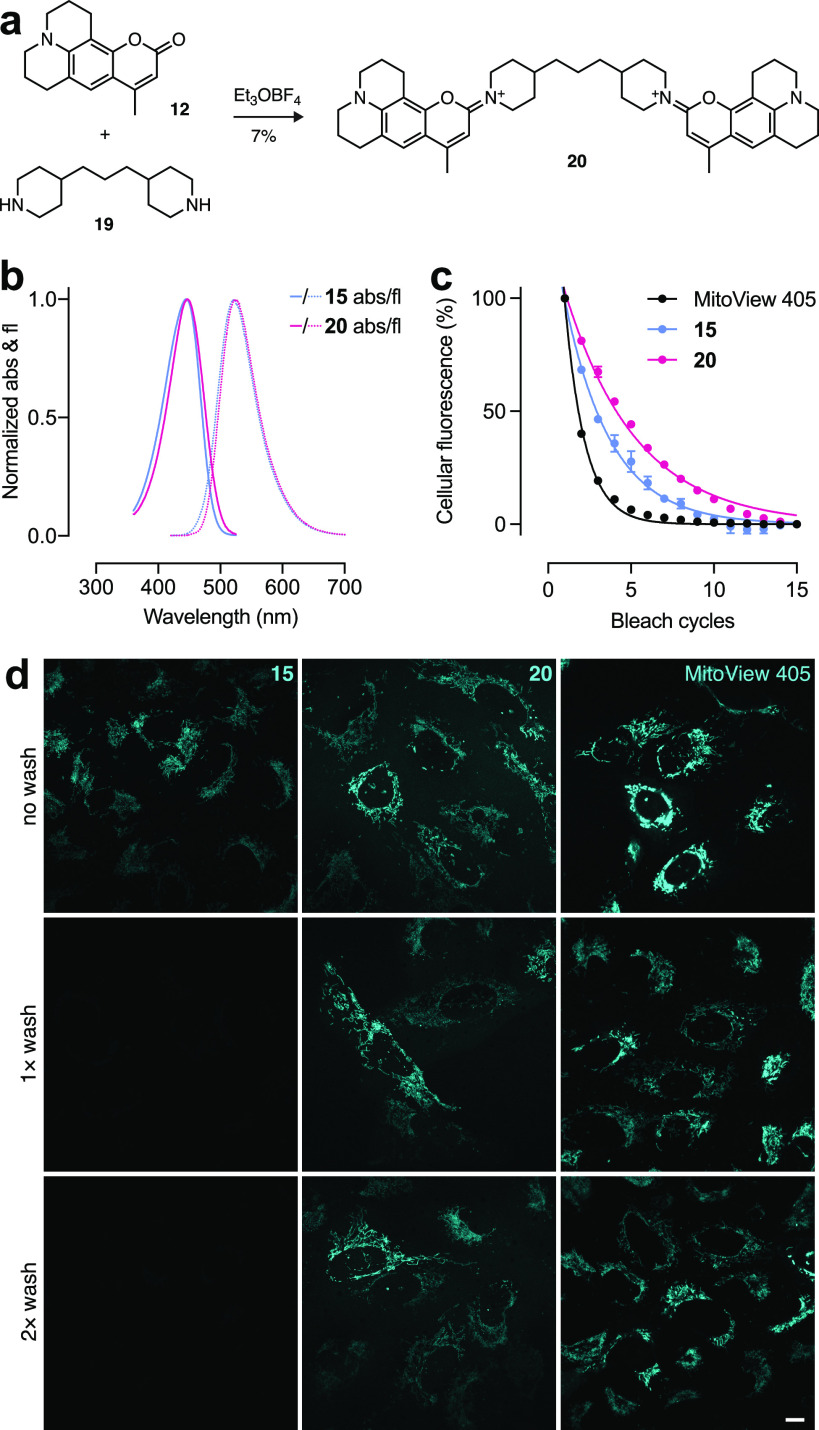
Dimerization of DAB dye **15** improves performance as a mitochondrial stain. (a) Synthesis of diDAB **20**. (b) Normalized absorption (abs) and fluorescence emission (fl) of **15** and **20**. (c) Plot of cellular fluorescence vs time of cells incubated with **15**, **20**, or MitoView 405 during photobleaching; error bars indicate SEM. (d) Live U2OS cells incubated with DAB **15** (200 nM), diDAB **20** (200 nM), or MitoView 405 (100 nM) after 0, 1, or 2 dye-free media exchange washes. Scale bar, 10 μm.

In these cellular imaging experiments, we noted that the cellular intensity of even the best DAB mitochondrial stain **15** rapidly decreased upon media exchange. To further improve this reagent, we prepared a dimer derivative of this molecule by reacting Coumarin 102 (**12**) with dipiperidine **19** to yield “diDAB” **20** (Figure 5a). This design is predicated on two concepts. First, the relatively long Stokes shift of the parent dye **15** (Table 1, Figure 2c) should minimize FRET between the two fluorophore units and preserve fluorescence quantum yield. Second, the presence of two cationic moieties per molecule of **20** should improve mitochondrial retention. Examination of the chemical properties of **20** revealed a slightly higher rate of hydrolysis relative to **15** in different pH conditions even when considering the presence of two iminium groups (Figure S5).

We then measured the spectral properties of the dye dimer. Compound **20** exhibited similar absorption (λ_abs_ = 447 nm) and fluorescence emission (λ_em_ = 524 nm) spectra compared to DAB monomer **15** (Figure 5b); both dyes showed a linear relationship between absorption and concentration ≤20 μM (Figure S6). In aqueous solution, the diDAB **20** did not show the expected 2-fold increase in absorptivity but exhibited ε = 45 200 M^–1^ cm^–1^ along with a modestly lower Φ_f_ = 0.45 (Table S1). To reconcile this observation and mimic the nonpolar environment of the mitochondrial inner membrane, we measured the spectral properties of compounds **15** and **20** in sodium dodecyl sulfate (SDS) micelles and dioxane/water mixtures (Table S1). We observed the expected higher ε = 78 900 M^–1^ cm^–1^ in PBS containing 0.1% w/v SDS along with a larger Φ_f_ = 0.83; compound **20** is also brighter in aqueous dioxane. Although dye **15** also showed higher absorptivity and fluorescence quantum yield in these nonpolar conditions relative to PBS, the effect was less pronounced. We postulate that the relatively low ε and Φ_f_ observed for compound **20** in aqueous solution is due, in part, to intramolecular interactions between the two chromophore units. These interactions could be reduced in the more hydrophobic environment of SDS micelles or dioxane/water mixtures, resulting in higher absorptivity and fluorescence quantum yield.

We then compared the parent DAB **15**, diDAB **20**, and the commercial violet-excited (and structurally mysterious) MitoView 405 in live-cell experiments. Although MitoView 405 was modestly brighter than DAB **15** or diDAB **20** upon initial application, the dye bleached rapidly in live cells in our hands, preventing acquisition of a full confocal microscopy stack. The DAB compounds exhibited substantially higher resistance to photobleaching with the diDAB **20** showing the best overall photostability (Figure 5c). As expected, all the dyes showed excellent mitochondrial staining upon initial application (Figure 5d). Upon media exchange, however, the DAB **15** signal rapidly decreased whereas the diDAB **20** and MitoView 405 were retained after this cell washing protocol.

## Conclusion

In summary, we demonstrate that the 2,7-diaminobenzopyrylium (DAB) framework is a modular scaffold for the synthesis of mitochondrial stains excited with violet light. These atom-efficient imaging reagents can be prepared from the broad palette of 7-aminocoumarin dyes with different N-substitution patterns (Scheme 1). Although the spectral properties can be tuned by choosing different coumarin starting materials, the structure of the secondary amine reactant has only minor effects on the properties of the resulting DAB dyes: use of azetidine or piperidine gave fluorophores with similar quantum yields and photostability (Table 1). This is in contrast to rhodamine dyes, where azetidine or piperidine auxochromes can elicit 10-fold changes in Φ_f_.^18^ The DAB dyes exhibit higher absorptivity and photostability than their coumarin parent dyes (Table 1, Figure 2), show reasonable chemical stability (Figure 3), and are effective mitochondrial stains (Figure 4); dimerization of the DAB dye affords a stain with better cellular retention (Figure 5). Looking forward, the utility of these bright, photostable, and biocompatible “mini-rhodamines” (Figure 1) could be expanded beyond mitochondrial stains. The stability of the iminium moiety could be improved through intramolecular cyclization to develop new conjugatable fluorescent labels or photolabile groups. The slow rate of hydrolysis could also be tuned and exploited to release coumarin-based drugs.^23^ Overall, the DAB dyes represent an underutilized chemical scaffold worthy of further attention.

## Supporting Information Available

Chemical stability of dyes in PBS, stability of **15** under different conditions, photochemistry of **15**, mitochondrial staining with DAB dyes, comparison of stability of **15** and **20**, Beer–Lambert–Bouguer analysis of **15** and **20**, spectral properties of **15** and **20** in different solvents, spectroscopy and imaging experimental details, synthetic organic chemistry details, and characterization of novel compounds (PDF)
