# Supplementary material for: 2,7-Diaminobenzopyrylium Dyes Are Live-Cell Mitochondrial Stains
Source: ACS Bio Med Chem Au. 2022 Feb 28;2(3):307–12. doi: 10.1021/acsbiomedchemau.1c00068 (PMC9204777; doi:10.1021/acsbiomedchemau.1c00068)
Supplement: Supplementary file 1 — bg1c00068_si_001.pdf [file bg1c00068_si_001.pdf]

SUPPORTING INFORMATION

2,7-Diaminobenzopyrylium dyes are live-cell mitochondrial stains

Sambashiva Banala, Ariana N. Tkachuk, Ronak Patel, Pratik Kumar, Timothy A. Brown, and Luke D. Lavis*

Janelia Research Campus, Howard Hughes Medical Institute, 19700 Helix Drive, Ashburn, VA 20147, USA

*Email: lavisl@janelia.hhmi.org

EXPERIMENTAL INFORMATION

Page	Contents
S2–S5	Figures S1–S6 and Table S1
S6	Experimental Details for Spectroscopy and Imaging
S9	Experimental Details and Characterization for Synthesis
S12	References
S13	NMR and LC–MS

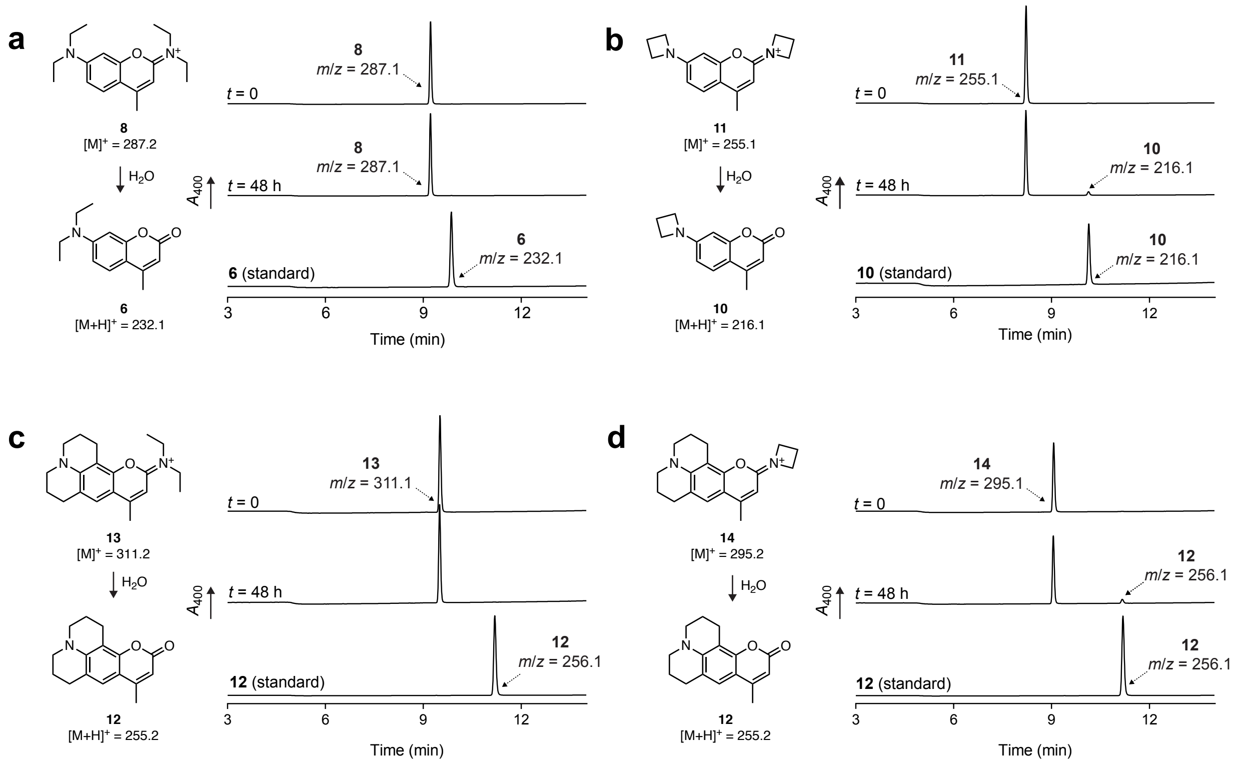

Figure S1. Chemical stability of DAB dyes 8**,** 11**, and** 13**–**14 **in PBS.** (**a**) Spontaneous hydrolysis of **8** to form coumarin **6** and LC–MS chromatograms of **8** at *t* = 0 (top), **8** at *t* = 48 h (middle), and **6** (bottom). (**b**) Spontaneous hydrolysis of **11** to form coumarin **10** and LC–MS chromatograms of **11** at *t* = 0 (top), **11** at *t* = 48 h (middle), and **10** (bottom). (**c**) Spontaneous hydrolysis of **13** to form coumarin **12** and LC–MS chromatograms of **13** at *t* = 0 (top), **13** at *t* = 48 h (middle), and **12** (bottom). (**d**) Spontaneous hydrolysis of **14** to form coumarin **12** and LC–MS chromatograms of **14** at *t* = 0 (top), **14** at *t* = 48 h (middle), and **12** (bottom).

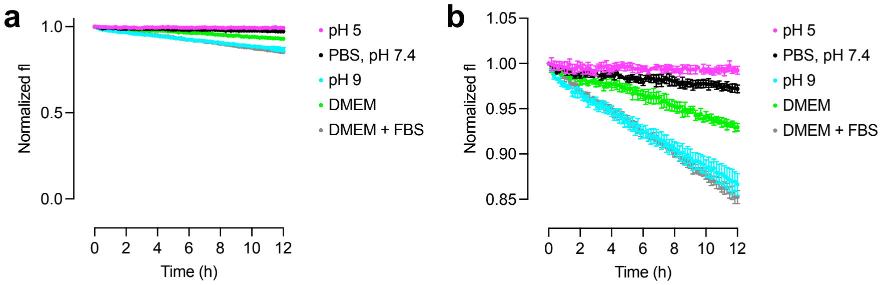

Figure S2. Chemical stability of DAB dye 15 **in different conditions.** Normalized fluorescence *vs.* time for compound **15** in the following conditions: citrate buffer, pH 5; PBS, pH 7.4; Tris buffer, pH 9; Dulbecco's Modified Eagle Medium (DMEM); and DMEM containing 10% v/v fetal bovine serum (DMEM + FBS). (**a**) Full plot. (**b**) Zoom-in of the ordinate to better show differences in hydrolysis rates. Error bars indicate SEM.

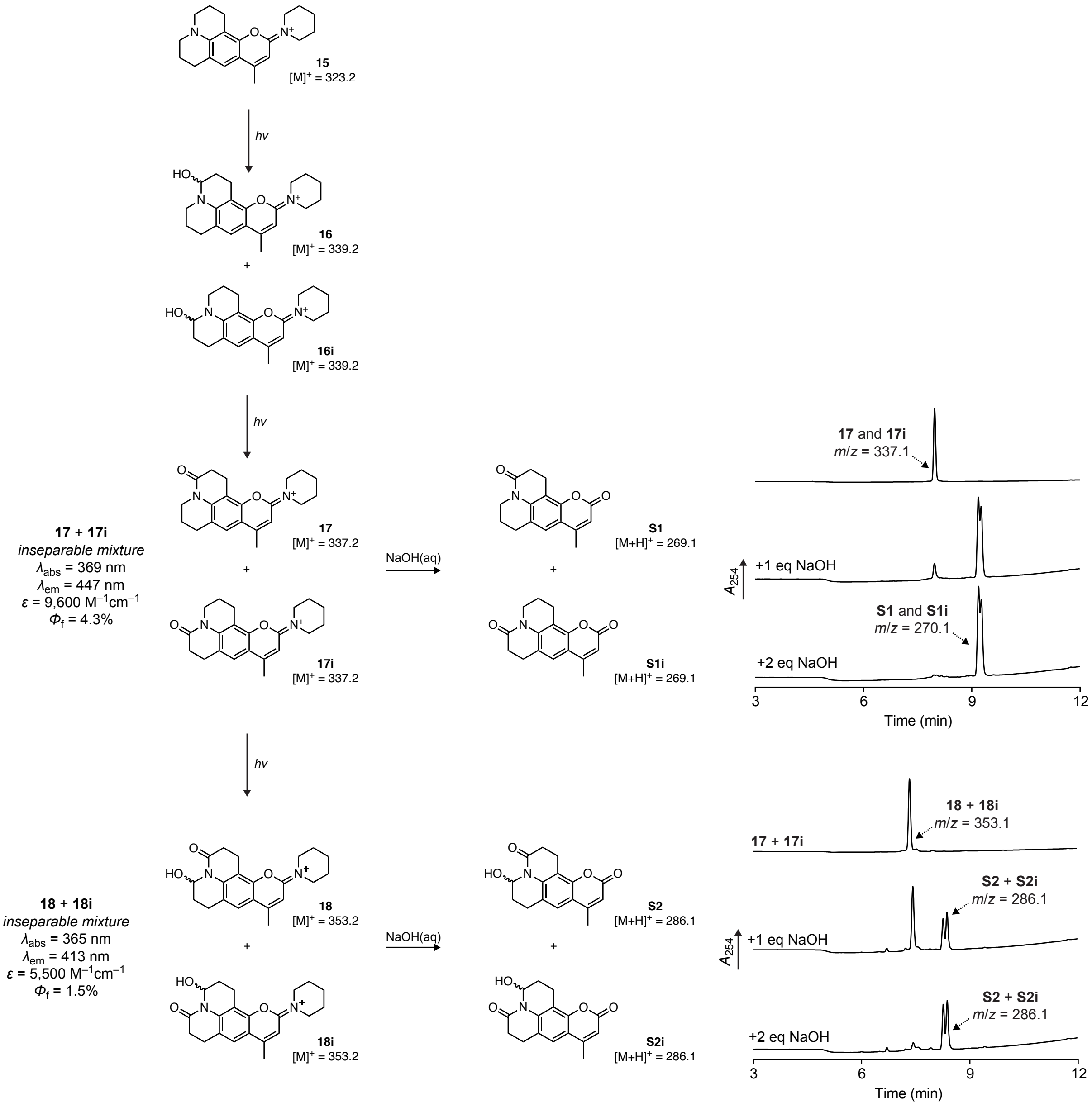

Figure S3. Photochemistry of DAB 15. Scheme showing the photochemical reactions of **15** to form **16**/**16i**, **17**/**17i**, and **18**/**18i**, spectral properties of oxidized products **17**/**17i** and **18**/**18i**, and hydrolysis products **S1**/**S1i** and **S2**/**S2i** with accompanying LC–MS traces confirming that photoinduced oxidation primarily occurs on the julolidine system.

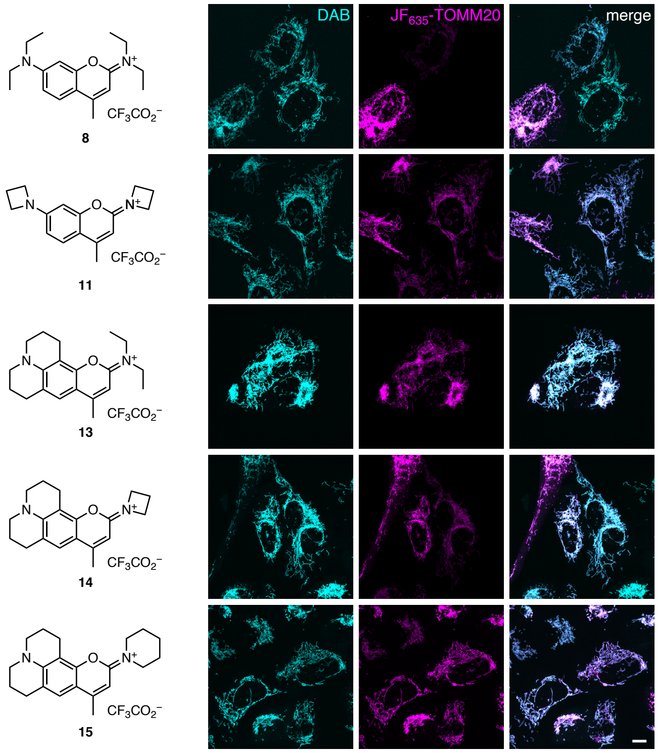

Figure S4. DAB dyes are mitochondrial stains. Chemical structures of DAB dyes **8**, **11**, and **13**–**15** and corresponding fluorescence images of U2OS cells transiently transfected with a plasmid encoding TOMM20–HaloTag fusion protein and incubated with JF_{635}–HaloTag ligand (200 nM) together with each DAB dye (200 nM) for 1 h at 37 °C. Scale bar: 10 µm. Note that the transient transfection of TOMM20–HaloTag results in a mixture of transfected and untransfected cells.

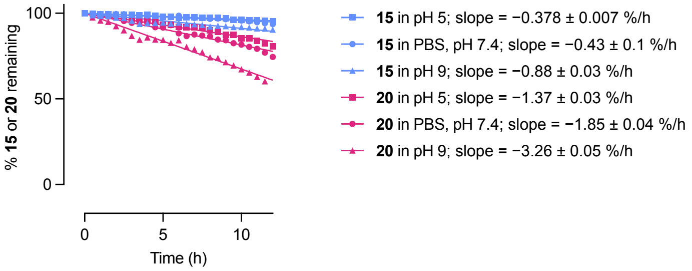

Figure S5. Comparison of the chemical stability of DAB dyes 15 **and** 20 **in different conditions.** Normalized percentage of compound **15** or **20** remaining *vs.* time for compound **15** in the following conditions: citrate buffer, pH 5; PBS, pH 7.4; Tris buffer, pH 9. The percentage of compound remaining is calculated from HPLC chromatograms taken every 30 min over 12 h. Slopes (± SEM) are calculated from linear regression of these points to allow quantitative comparison of the differences in initial hydrolysis rates.

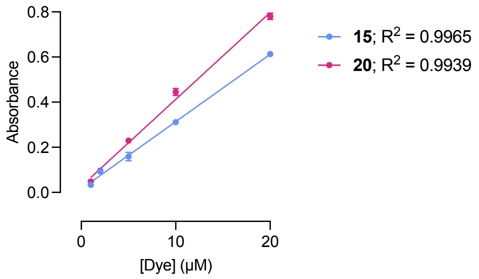

Figure S6. Beer–Lambert–Bouguer analysis of DAB dyes 15 **and** 20**.** Absorbance *vs.* dye concentration for compounds **15** and **20** in PBS, pH 7.4. Error bars indicate SD. R^2 values are from linear regression analysis.

Table S1. Spectral properties of DAB **15** and diDAB **20** in different solvents.

scaffold	solvent	λ_{abs} (nm)	ε ($M^{-1}cm^{-1}$)	λ_{em} (nm)	Φ_f
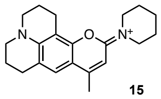 **15**	PBS	446	34,600	522	0.66
			34%		23%
	PBS + 0.1% SDS	453	46,200	525	0.81
	1:1 dioxane:H_2O	452	41,000	523	0.86
	9:1 dioxane:H_2O	450	40,900	526	0.88
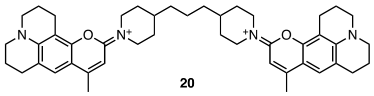 **20**	PBS	447	45,200	524	0.45
			75%		84%
	PBS + 0.1% SDS	455	78,900	525	0.83
	1:1 dioxane:H_2O	455	68,300	524	0.84
	9:1 dioxane:H_2O	450	69,100	526	0.86

Experimental Information for Spectroscopy and Imaging

Compound sources. Coumarins **6** (Coumarin 1) and **12** (Coumarin 102) were purchased from Exciton. Compound **10** was available from previous work.[1]

Buffers *etc.* All solutions were prepared using purified water from a Milli-Q Synthesis water purification system (Millipore). Phosphate-buffered saline (PBS), pH 7.4 was prepared from a 10× stock (Corning, 46-013-CM). Citrate buffer, pH 5 contained 10 mM citrate and 150 mM NaCl. Tris buffer, pH 9 was contained 10 mM tris(hydroxymethyl)aminomethane and 150 mM NaCl. To prepare sodium dodecyl sulfate (SDS) micelles, ultrapure SDS (J.T. Baker, 4095-04) was added directly to PBS at 1 mg/mL. Dioxane:water mixtures were prepared using anhydrous dioxane (Sigma-Aldrich, 296309). Dulbecco's Modified Eagle Medium (DMEM, phenol red-free) and fetal bovine serum (FBS) were obtained from Life Technologies.

UV–vis and fluorescence spectroscopy (Table 1, Figure 2a–c, Figure 5b, Figure S6, Table S1). Fluorescent molecules for spectroscopy were prepared as stock solutions in DMSO and diluted such that the DMSO concentration did not exceed 1% v/v. Spectroscopy was performed using 1-cm path length, 3.5-mL quartz cuvettes from Starna Cells. All measurements were taken at ambient temperature (22 ± 2 °C). Absorption spectra were recorded on a Cary Model 100 spectrometer (Agilent). Fluorescence spectra were recorded on a Cary Eclipse fluorometer (Varian). Unless otherwise noted, maximum absorption wavelength (λ_{abs}), extinction coefficient (ε), and maximum emission wavelength (λ_{em}) were measured in PBS, pH 7.4. Reported values and spectra are averages ($n = 3$). Normalized spectra are shown for clarity. For the Beer–Lambert–Bouguer analysis of DAB dyes **15** and **20**, 100× DMSO stock solutions of dyes were prepared to ensure consistent [DMSO] = 1%v/v ; absorption was measured at the λ_{abs} ($n = 3$).

Quantum yield determination (Table 1, Table S1). All reported absolute fluorescence quantum yield values (Φ_f) were measured in our laboratory under identical conditions using a Quantaurus-QY spectrometer (model C11374, Hamamatsu). Unless otherwise noted, Φ_f values were measured in PBS, pH 7.4. This instrument uses an integrating sphere to determine photons absorbed and emitted by a sample. Measurements were carried out using dilute samples ($A < 0.1$) and self-absorption corrections[2] were performed using the instrument software. Reported values are averages ($n = 3$).

***In vitro* photobleaching measurements (Figure 2d–f).** Solutions of **8**, **11**, **13**–**15** (5 µM) were prepared in 10 mM HEPES, pH 7.3. An aliquot of these solutions was added to each well of 10-well Teflon printed glass slide with 1.5 mm well diameter (Tekdon, Inc.) and sealed with a coverslip using vacuum grease. The hydrophobic coating of the slide resulted in formation of macrodroplets of aqueous dye solution in each well. Fluorophore bleaching was measured by illuminating an entire droplet using an upright microscope (Zeiss Axio Observer Z2) and a 5×/0.25 NA objective. Light illumination was provided by a mercury lamp (X-Cite Series 120-Q) with two filter sets depending on dye type. For the coumarin dyes, the light was passed through an excitation filter centered at 390 nm with a 40 nm

band-pass at 2.8 W/cm^2 intensity. For the DAB dyes, the light was passed through an excitation filter centered at 435 nm with a 40 nm band-pass at 4.55 W/cm^2 intensity. Fluorescence emission was collected through a 488 nm long-pass filter and detected with a fiber-coupled avalanche photodiode detector (SPQM-AQRH14; Pacer); n = 3.

The obtained bleaching profiles were fit using a single exponential fitting function in MATLAB program to calculate their respective time constants (t_b). To allow a quantitative comparison of photobleaching for different fluorophores, the number of photons (N_p) emitted per molecule before photobleaching was calculated as previously described.[3] Briefly, the N_p for each fluorophore was quantified from their respective excitation rate (W) and fluorescence quantum yield (Φ_f). The excitation rate W (s^{-1}) was calculated by integrating the product of extinction coefficient (ε_λ; in M^{-1}·cm^{-1}) and intensity (I_λ; in W·cm^{-2}) for the discrete wavelength (λ; in µm) as shown in Equation 1:

$$W = 0.0192 \sum \varepsilon_\lambda \cdot I_\lambda \cdot \lambda \quad (1)$$

Photobleaching was characterized by calculating the number of photons (N_p) as the product of fluorescence quantum yield (Φ_f), excitation rate (W) and photobleaching time constant (t_b) as shown in Equation 2:

$$N_p = \Phi_f \cdot W \cdot t_b \quad (2)$$

Measurement of chemical stability and photostability by tandem high-pressure liquid chromatography–mass spectrometry (LC–MS; Figure 3, Figure S1, Figure S3, Figure S5). To examine the chemical stability and photostability of the DAB dyes, we assessed samples by tandem high-pressure liquid chromatography–mass spectrometry (LC–MS) using an Agilent 1200 LC–MS system equipped with an autosampler, diode array detector, and mass spectrometry detector (ESI; positive ion mode) using a 4.6 × 150 mm Gemini NX-C18 column with a 5–95% or 5–50% gradient of CH_3CN in H_2O containing constant 0.1% (v/v) TFA. Chromatograms were monitored using absorbance at 254 nm or 400 nm. To investigate chemical hydrolysis, solutions of **8**, **11**, and **13**–**15** (100 µM) were prepared in PBS, pH 7.4. An aliquot of this freshly prepared solution was immediately analyzed by LC–MS (t = 0). The solution was then incubated at ambient temperature protected from light for 48 h and analyzed again by LC–MS (t = 48 h). Standard samples of coumarins **6**, **10**, and **12** (100 µM, PBS) were analyzed in the same manner. To compare the stability of compounds **15** and **20** under different conditions, solutions of each dye (25 µM) in citrate buffer, pH 5, PBS, pH 7.4, or Tris buffer, pH 9 were prepared in an autosampler vial and the sample was analyzed using the LC–MS instrumentation every 30 min. We note that the autosampler chamber in this LC–MS system is not temperature controlled and the higher rates of hydrolysis measured using this method reflect incubation of the solutions at higher-than-ambient temperature.

To investigate photochemistry, solutions of **8**, **11**, and **13**–**15** (100 µM) were prepared in PBS and placed in a glass vial. An aliquot of this freshly prepared solution was immediately analyzed by LC–MS ($-h\nu$). The solution was then irradiated with 405 nm light from an LED array (LOCTITE CL20 flood array) for 5 min (t = 5 min) and 20 min (t = 20 min) followed by analysis using LC–MS. Photochemical adducts **17**/**17i** and **18**/**18i** were purified and the spectral properties of these isomeric mixtures were analyzed. These compounds were then treated with 1 or 2 equivalents of NaOH(aq) to hydrolyze the iminium bond; the resulting oxidized coumarin products **S1**/**S1i** and **S2**/**S2i** were observed by LC–MS.

Measurement of chemical stability by fluorescence (Figure S2). To compare the chemical stability of **15** in different conditions using changes in fluorescence, we prepared samples (1 μM) of **15** in the following solutions: citrate buffer, pH 5; PBS, pH 7.4; Tris buffer, pH 9; DMEM; and DMEM containing 10% v/v FBS (DMEM + FBS). 200 μL of each solution (n = 3) was placed in a black, clear-bottom 96-well microplate (Nunc, 165305) and sealed with an adhesive film. The fluorescence of the DAB dye was monitored using 445 nm excitation and 510–550 nm emission (integrated signal) reading from the bottom of the plate using a TECAN Spark microplate reader controlled with SparkControl software.

Cell culture and confocal fluorescence microscopy (Figure 4, Figure 5d, Figure S4). U2OS cells (ATCC) were cultured in Dulbecco's modified Eagle medium (DMEM, phenol red-free; Life Technologies) supplemented with 10% v/v fetal bovine serum (FBS, Life Technologies), 1 mM GlutaMAX (Life Technologies) and maintained at 37 °C in a humidified 5% (v/v) CO_2 environment. This cell line undergoes regular mycoplasma testing by the Janelia Cell Culture Facility. Live-cell microscopic imaging was performed on a Zeiss LSM 800 or 880 confocal microscope with a Plan APO 63×/1.4 oil DIC M27 objective. For co-staining mitochondria using MitoTracker Deep Red, live U2OS cells were first incubated with MitoTracker Deep Red (100 nM; ThermoFisher) for 15 min at 37 °C, washed 2× with dye-free media then incubated with DAB dyes **8**, **11**, **13**–**15** (200 nM) for 1 h at 37 °C and imaged live without washing. For co-staining mitochondria using TOMM20-HaloTag labeling, U2OS cells were transiently transfected with a plasmid expressing a TOMM20–HaloTag fusion protein by nucleofection (Lonza); TOMM20 is an outer mitochondrial membrane protein as part of a protein translocase complex. Cells were incubated with JF_{635}–HaloTag ligand (200 nM) together with each DAB dye (200 nM) for 1 h at 37 °C and imaged live without washing. The confocal image stacks were processed using FIJI[4] and displayed as maximum intensity image projections.

To compare cellular retention of **15**, **20**, and MitoView 405, U2OS cells were incubated with **15** (200 nM), **20** (200 nM) or MitoView 405 (100 nM; Biotium) for 1 h at 37 °C. These samples were imaged live without washing, after 1× wash with dye-free media, or after 2× wash with dye-free media. The confocal images were processed using FIJI[4] and displayed as maximum intensity image projections.

In-cell photostability experiments (Figure 5c). U2OS cells were incubated with **15** (200 nM), **20** (200 nM) or MitoView 405 (100 nM; Biotium) for 1 h at 37 °C and imaged live without washing using a Leica SP8 Falcon confocal microscope with enabled adaptive focus control (AFC), an HC PL-APO 86×/1.20 water objective, and a 405 nm diode laser at 58.9% laser power. Imaging was continued over 15 photobleaching cycles; n = 3. These images were processed using FIJI.[4]

Statistics and Reproducibility. For spectroscopy measurements and photobleaching experiments, reported n values represent measurements of different samples prepared from the same dye DMSO stock solution. For cell-based photostability experiments, n represents different fields of view. For fluorescence microscopy imaging experiments, all procedures were repeated at least once on a separate biological sample to ensure results were similar.

Experimental Information for Synthesis

General. Commercial reagents were obtained from reputable suppliers and used as received. All solvents were purchased in septum-sealed bottles stored under an inert atmosphere. Azetidinylcoumarin **10** was synthesized as previously described.[1] All reactions were sealed with septa through which an argon atmosphere was introduced unless otherwise noted. Reactions were conducted in round-bottomed flasks or septum-capped crimp-top vials containing Teflon-coated magnetic stir bars. Heating of reactions was accomplished with an aluminum reaction block on top of a stirring hotplate equipped with an electronic contact thermometer to maintain the indicated temperatures.

Reactions were monitored by thin layer chromatography (TLC) on precoated TLC glass plates (silica gel 60 F_{254}, 250 µm thickness) or by LC–MS (Phenomenex Kinetex 2.1 mm × 30 mm 2.6 µm C18 column; 5 µL injection; 5–98% $MeCN/H_2O$, linear gradient, with constant 0.1% v/v HCO_2H additive; 6 min run; 0.5 mL/min flow; ESI; positive ion mode). TLC chromatograms were visualized by UV illumination or developed with *p*-anisaldehyde, ceric ammonium molybdate, or $KMnO_4$ stain. Reaction products were purified by preparative HPLC (Phenomenex Gemini–NX 30 × 150 mm 5 µm C18 column). Tandem high-pressure liquid chromatography–mass spectrometry (LC–MS) was performed on an Agilent 1200 LC–MS system equipped with an autosampler, diode array detector, and mass spectrometry detector (ESI; positive ion mode) using a 4.6 × 150 mm Gemini NX-C18 column with a 5–95% or 5–50% gradient of CH_3CN in H_2O containing constant 0.1% (v/v) trifluoroacetic acid (TFA). High-resolution mass spectrometry was performed by the High Resolution Mass Spectrometry Facility at the University of Iowa.

NMR spectra were recorded on a 400 MHz spectrometer. ^1H and ^{13}C chemical shifts were referenced to TMS or residual solvent peaks. Data for ^1H NMR spectra are reported as follows: chemical shift (δ ppm), multiplicity (s = singlet, d = doublet, t = triplet, q = quartet, dd = doublet of doublets, m = multiplet), coupling constant (Hz), integration. Data for ^{13}C NMR spectra are reported by chemical shift (δ ppm) with hydrogen multiplicity (C, CH, CH_2, CH_3) information obtained from DEPT spectra. To determine the equivalents of TFA in the preparative HPLC-purified products, an internal fluorobenzene standard was added to **8**, **11**, **13**–**15**, or **20** in $CDCl_3$ or CD_3CN. Integration of the ^1H NMR and ^{19}F NMR signals from the fluorobenzene and DAB dyes allowed calculation of TFA equivalents. We found **8**, **11**, and **13**–**15** contain 4 equivalents of TFA per molecule and the dimer **20** contains 8 equivalents of TFA per molecule.

2,7-Bis(diethylamino)-4-methylbenzopyrylium trifluoroacetate (8): The following procedure for dye (**8**) is representative. To a solution of 7-diethylamino-4-methylcoumarin (Coumarin 1, **6**; 1.0 g, 4.32 mmol) in 20 mL of anhydrous CH_2Cl_2 was added triethyloxonium tetrafluoroborate (4.32 mmol, 4.3 mL of a 1 M solution in CH_2Cl_2, 1 equiv). The reaction was stirred for 40 min at 30 °C after which diethylamine (2 mL, 20 mmol, 4.63 equiv) was added and the reaction heated to reflux for 2 h. The reaction was cooled to room temperature and the reaction was partitioned between water and CH_2Cl_2. The organic solution was separated and washed with water, saturated NaCl(aq), and dried over $MgSO_4$. This was filtered and concentrated under reduced pressure. The residue was dissolved in CH_2Cl_2 (6 mL)

after which diethyl ether (30 mL) was added. The resulting precipitate was collected by filtration and partially purified by crystallization from hexane:acetone (1:1). This product was further purified by reverse-phase preparative HPLC using 5→95% v/v CH_3CN in H_2O, linear gradient with a constant 0.1% v/v TFA additive. The product-containing fractions were lyophilized, affording the desired product **8** (710 mg, 41%) as yellow powder. ^1H NMR ($CDCl_3$, 400 MHz) δ 7.60 (d, *J* = 9.2 Hz, 1H), 6.81 (dd, *J* = 9.2, 2.5 Hz, 1H), 6.62 (d, *J* = 2.6 Hz,, 1H) 6.54 (s, 1H), 3.83 (q, *J* = 7.2 Hz, 2H), 3.75 (q, *J* = 7.2 Hz, 2H), 3.50 (q, *J* = 7.1 Hz, 4H), 2.58 (s, 3H), 1.43–3.50 (m, 6H), 1.26 (t, *J* = 7.1 Hz, 6H). ^{13}C NMR ($CDCl_3$, 101 MHz) δ 161.1 (C), 158.6 (C), 154.7 (C), 152.9 (C), 127.2 (CH), 112.1 (CH), 109.3 (C), 99.9 (CH), 96.4 (CH), 45.6 (CH_2), 45.2 (CH_2), 44.9 (CH_2), 19.4 (CH_3), 13.1 (CH_3), 12.7 (CH_3), 12.4 (CH_3). HRMS (ESI) calcd for $C_{18}H_{27}N_2O$ $[M]^+$ 287.2118, found 287.2114.

2,7-Di(azetidin-1-yl) 4-methylbenzopyrylium trifluoroacetate (11): This compound was prepared using azetidinylcoumarin[1] **10** and azetidine according to the general procedure described for compound **8**. Method for reverse-phase HPLC: 5→95% v/v CH_3CN in H_2O, linear gradient, with constant 0.1% v/v TFA additive. Yield: 24% as a pale yellow powder. ^1H NMR ($CDCl_3$, 400 MHz) δ 7.54 (d, *J* = 8.8 Hz, 1H), 6.44 (dd, *J* = 8.9, 2.2 Hz, 1H), 6.29 (d, *J* = 2.2 Hz, 1H), 6.24 (s, 1H), 4.65–4.54 (m, 4H), 4.11 (t, *J* = 7.5 Hz, 4H), 2.67 (p, *J* = 7.9 Hz, 2H), 2.55–2.45 (m, 5H). ^{13}C NMR ($CDCl_3$, 101 MHz) δ 160.4 (C), 157.7 (C), 154.8 (C), 154.0 (C), 127.1 (CH), 110.4 (CH), 109.9 (C), 99.7 (CH), 95.7 (CH), 52.6 (CH_2), 52.1 (CH_2), 51.6 (CH_2), 19.3 (CH_3), 16.6 (CH_2), 16.4 (CH_2). HRMS (ESI) calcd for $C_{16}H_{19}N_2O$ $[M]^+$ 255.1492, found 255.1488.

***N*-ethyl-*N*-(9-methyl-2,3,6,7-tetrahydro-1*H*,5*H*,11*H*-pyrano[2,3-*f*]pyrido[3,2,1-*ij*]quinolin-11-ylidene)ethanaminium trifluoroacetate (13):** This compound was prepared using Coumarin 102 (**12**) and diethylamine according to the general procedure described above for compound **8**. Method for reverse-phase preparative HPLC: 5→95% v/v CH_3CN in H_2O, linear gradient, with constant 0.1% v/v TFA additive. Yield: 43% as a yellow powder. ^1H NMR ($CDCl_3$, 400 MHz) δ 7.19 (s, 1H), 6.40 (s, 1H), 3.78 (q, *J* = 7.2 Hz, 2H), 3.71 (q, *J* = 7.2 Hz, 2H), 3.41–3.34 (m, 4H), 2.86 (t, *J* = 6.4 Hz, 2H), 2.82 (t, *J* = 6.5 Hz, 2H), 2.54 (s, 3H), 2.08–1.95 (m, 4H), 1.43–1.33 (m, 6H). ^{13}C NMR ($CDCl_3$, 101 MHz) δ 160.7 (C), 158.4 (C), 149.8 (C), 148.5 (C), 123.1 (CH), 121.9 (C), 109.2 (C), 105.5 (C), 99.0 (CH), 50.3 (CH_2), 49.6 (CH_3), 27.9 (CH_3), 20.9 (CH_2), 20.1 (CH_2), 20.01 (CH_2), 19.5 (CH_3). HRMS (ESI) calcd for $C_{20}H_{27}N_2O$ $[M]^+$ 311.2118 found 311.2112.

1-(9-methyl-2,3,6,7-tetrahydro-1H,5H,11*H*-pyrano[2,3-f]pyrido[3,2,1-ij]quinolin-11-ylidene)azetidin-1-ium trifluoroacetate (**14**): This compound was prepared using Coumarin 102 (**12**) and azetidine according to the general procedure described above for compound **8**. Method for reverse-phase preparative HPLC: 5→95% v/v CH_3CN in H_2O, linear gradient, with constant 0.1% v/v TFA additive. Yield: 10% as a yellow powder. ^1H NMR ($CDCl_3$, 400 MHz) δ 7.15 (s, 1H), 6.08 (s, 1H), 4.56 (t, *J* = 7.8 Hz, 4H), 3.40 – 3.31 (m, 4H), 2.81 (t, *J* = 6.3 Hz, 4H), 2.67 (p, *J* = 7.8 Hz, 2H), 2.48 (s, 3H), 2.06 – 1.92 (m, 4H). ^{13}C NMR ($CDCl_3$, 101 MHz) δ 160.3 (C), 157.4 (C), 149.4 (C), 148.3 (C), 123.1 (CH), 121.5 (C), 109.1 (C), 105.9 (C), 98.0 (CH), 52.1 (CH_2), 51.4 (CH_2), 50.2 (CH_2), 49.7 (CH_2), 27.9

(CH_2), 21.0 (CH_2), 20.0 (CH_2), 19.9 (CH_2), 19.1 (CH_3), 16.6 (CH_2). HRMS (ESI) calcd for $C_{19}H_{23}N_2O$ $[M]^+$ 295.1805 found 295.1801.

1-(9-methyl-2,3,6,7-tetrahydro-1*H*,5*H*,11*H*-pyrano[2,3-*f*]pyrido[3,2,1-*ij*]quinolin-11-ylidene)piperidin-1-ium trifluoroacetate (**15**): This compound was prepared using Coumarin 102 (**12**) and piperidine according to the general procedure described above for compound **8**. Method for reverse-phase preparative HPLC: 5→95% v/v CH_3CN in H_2O, linear gradient, with constant 0.1% v/v TFA additive. Yield: 45% as a yellow powder. ^1H NMR ($CDCl_3$, 400 MHz) δ 7.16 (s, 1H), 6.51 (s, 1H), 4.01–3.67 (m, 4H), 3.45–3.29 (m, 4H), 2.87–2.76 (m, 4H), 2.51 (s, 3H), 2.09–1.89 (m, 4H), 1.86–1.73 (m, 6H). ^{13}C NMR ($CDCl_3$, 101 MHz) δ 159.8 (C), 158.4 (C), 149.5 (C), 148.6 (C), 122.9 (CH), 121.9 (C), 109.0 (C), 105.5 (C), 98.7 (CH), 50.2 (CH_2), 49.6 (CH_2), 27.8 (CH_2), 25.7 (CH_2), 23.5 (CH_2), 20.9 (CH_2), 20.3 (CH_2), 19.9 (CH_2), 19.4 (CH_3). HRMS (ESI) calcd for $C_{21}H_{27}N_2O$ $[M]^+$ 323.2118 found 323.2111.

4,4'-(propane-1,3-diyl)bis(1-(9-methyl-2,3,6,7-tetrahydro-1*H*,5*H*,11*H*-pyrano[2,3-*f*]pyrido[3,2,1-*ij*]quinolin-11-ylidene)piperidin-1-ium) trifluoroacetate (**20**): To a solution of Coumarin 102 (**12**; 255 mg, 1.0 mmol, 10 equiv) in 20 mL of anhydrous CH_2Cl_2 was added triethyloxonium tetrafluoroborate (1.0 mmol, 1.0 mL of 1 M in CH_2Cl_2, 10 equiv). The reaction was stirred for 40 min at 30 °C, after which 4,4′-trimethylenedipiperidine (**16**, 21 mg, 0.1 mmol, 1 equiv) was added and the reaction was heated to reflux for 48 h. The reaction was cooled to room temperature and the reaction was partitioned between water and CH_2Cl_2. The organic solution was separated and washed with water, saturated NaCl(aq), and dried over $MgSO_4$. This was filtered and concentrated under reduced pressure. The resulting precipitate was collected by filtration and partially purified by crystallization from hexane:acetone (1:1). This product was further purified by reverse-phase preparative HPLC using 5→90% v/v CH_3CN in H_2O, linear gradient with a constant 0.1% v/v TFA additive. The product-containing fractions were lyophilized, affording the desired product **20** (6 mg, 7%) as yellow powder. ^1H NMR ($CDCl_3$, 400 MHz) δ 7.15 (s, 2H), 6.59 (s, 2H), 4.68–4.52 (m, 2H), 4.38–4.20 (m, 2H), 3.45–3.21 (m, 12H), 2.91–2.76 (m, 8H), 2.52 (s, 6H), 2.10–1.91 (m, 12H), 1.81–1.66 (m, 2H). 1.43–1.25 (m, 10H). ^{13}C NMR ($CDCl_3$, 101 MHz) δ 159.8 (C), 158.3 (C), 149.5 (C), 148.5 (C), 122.9 (CH), 121.8 (C), 109.1 (C), 105.7 (C), 99.1 (CH), 50.3 (CH_2), 49.7 (CH_2), 47.7 (CH_2), 46.0 (CH_2), 35.5 (CH_2), 34.8 (CH), 31.8 (CH_2), 27.9 (CH_2), 23.1 (CH_2), 21.0 (CH_2), 20.3 (CH_2), 20.0 (CH_2), 19.5 (CH_3)HRMS (ESI) calcd for $C_{45}H_{58}N_4O_2$ $[M]^{2+}$ 343.2274 found 343.2275.

References

1. Grimm, J. B.; English, B. P.; Chen, J.; Slaughter, J. P.; Zhang, Z.; Revyakin, A.; Patel, R.; Macklin, J. J.; Normanno, D.; Singer, R. H.; Lionnet, T.; Lavis, L. D., A general method to improve fluorophores for live-cell and single-molecule microscopy. *Nat. Methods* **2015,** *12* (1548-7105 (Electronic)), 244–250.
2. Suzuki, K.; Kobayashi, A.; Kaneko, S.; Takehira, K.; Yoshihara, T.; Ishida, H.; Shiina, Y.; Oishi, S.; Tobita, S., Reevaluation of absolute luminescence quantum yields of standard solutions using a spectrometer with an integrating sphere and a back-thinned CCD detector. *Phys. Chem. Chem. Phys.* **2009,** *11* (42), 9850-9860.
3. Abdelfattah, A. S.; Kawashima, T.; Singh, A.; Novak, O.; Liu, H.; Shuai, Y.; Huang, Y. C.; Campagnola, L.; Seeman, S. C.; Yu, J.; Zheng, J.; Grimm, J. B.; Patel, R.; Friedrich, J.; Mensh, B. D.; Paninski, L.; Macklin, J. J.; Murphy, G. J.; Podgorski, K.; Lin, B. J.; Chen, T. W.; Turner, G. C.; Liu, Z.; Koyama, M.; Svoboda, K.; Ahrens, M. B.; Lavis, L. D.; Schreiter, E. R., Bright and photostable chemigenetic indicators for extended in vivo voltage imaging. *Science* **2019,** *365* (6454), 699-704.
4. Schindelin, J.; Arganda-Carreras, I.; Frise, E.; Kaynig, V.; Longair, M.; Pietzsch, T.; Preibisch, S.; Rueden, C.; Saalfeld, S.; Schmid, B.; Tinevez, J. Y.; White, D. J.; Hartenstein, V.; Eliceiri, K.; Tomancak, P.; Cardona, A., Fiji: An open-source platform for biological-image analysis. *Nat. Methods* **2012,** *9* (7), 676-682.

Origin	Bruker BioSpin GmbH
Solvent	CDCl3
Temperature	300.0
Pulse Sequence	zg30
Experiment	1D
Number of Scans	16
Spectrometer Frequency	400.13
Spectral Width	8012.8
Lowest Frequency	-1545.5
Nucleus	1H
Acquired Size	32768
Spectral Size	65536

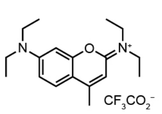

8

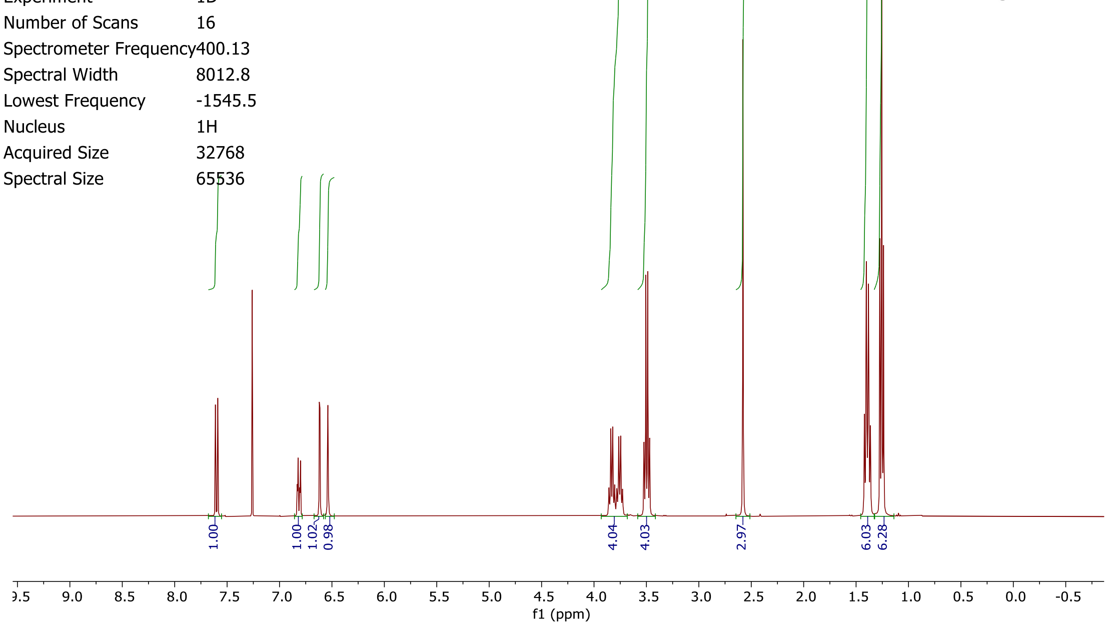

Origin	Bruker BioSpin GmbH
Solvent	CDCl3
Temperature	300.0
Pulse Sequence	zgpg30
Experiment	1D
Number of Scans	5120
Spectrometer Frequency	100.62
Spectral Width	24038.5
Lowest Frequency	-1945.4
Nucleus	13C
Acquired Size	32768
Spectral Size	65536

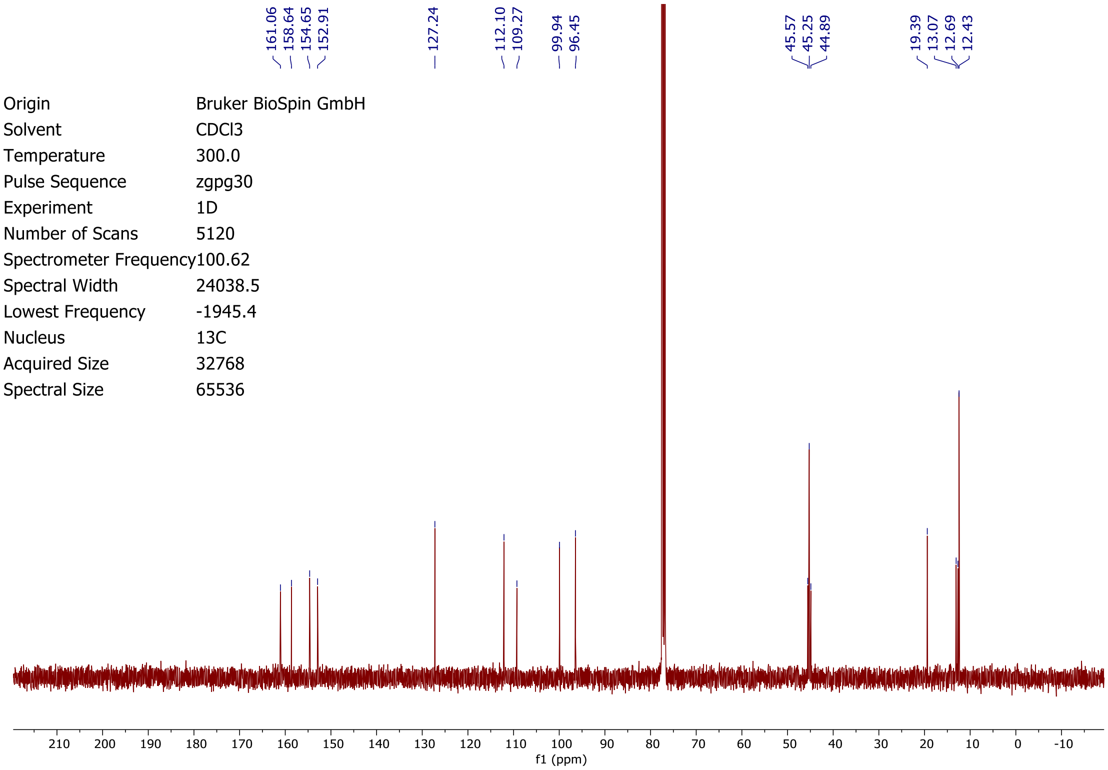

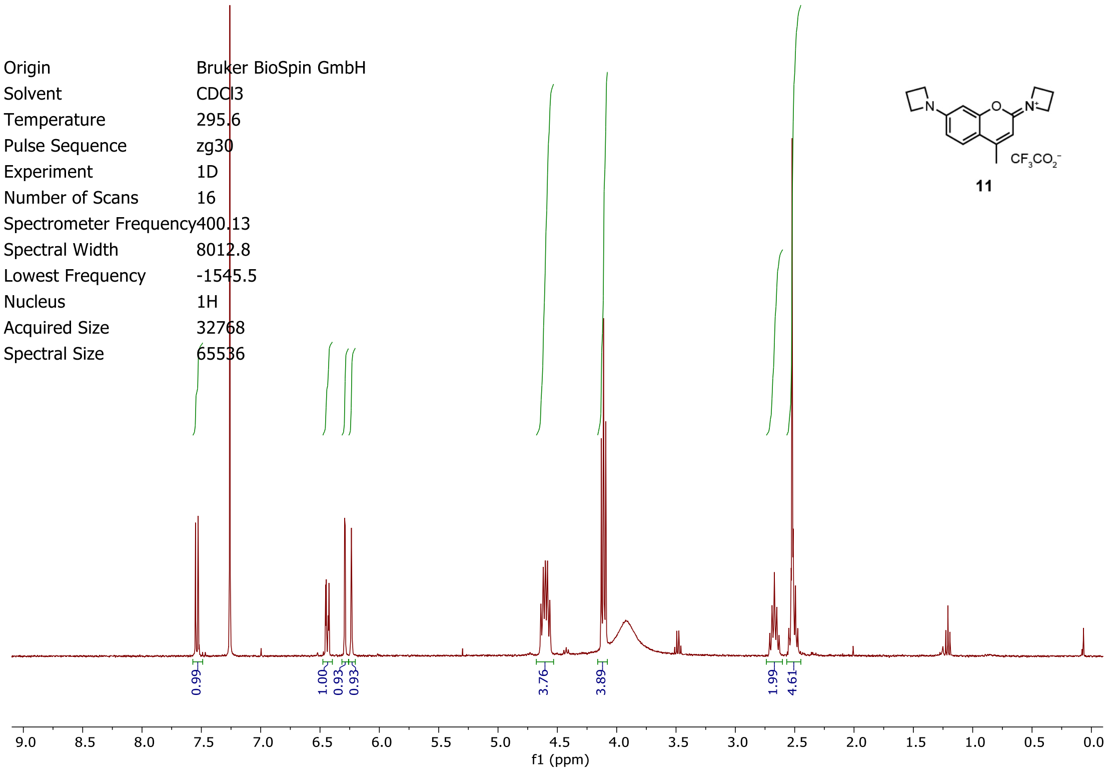

11
Origin Bruker BioSpin GmbH
Solvent CDCl3
Temperature 295.6
Pulse Sequence zg30
Experiment 1D
Number of Scans 16
Spectrometer Frequency 400.13
Spectral Width 8012.8
Lowest Frequency -1545.5
Nucleus 1H
Acquired Size 32768
Spectral Size 65536
0.99
1.00
0.93
0.93
3.76
3.89
1.99
4.61
f1 (ppm)

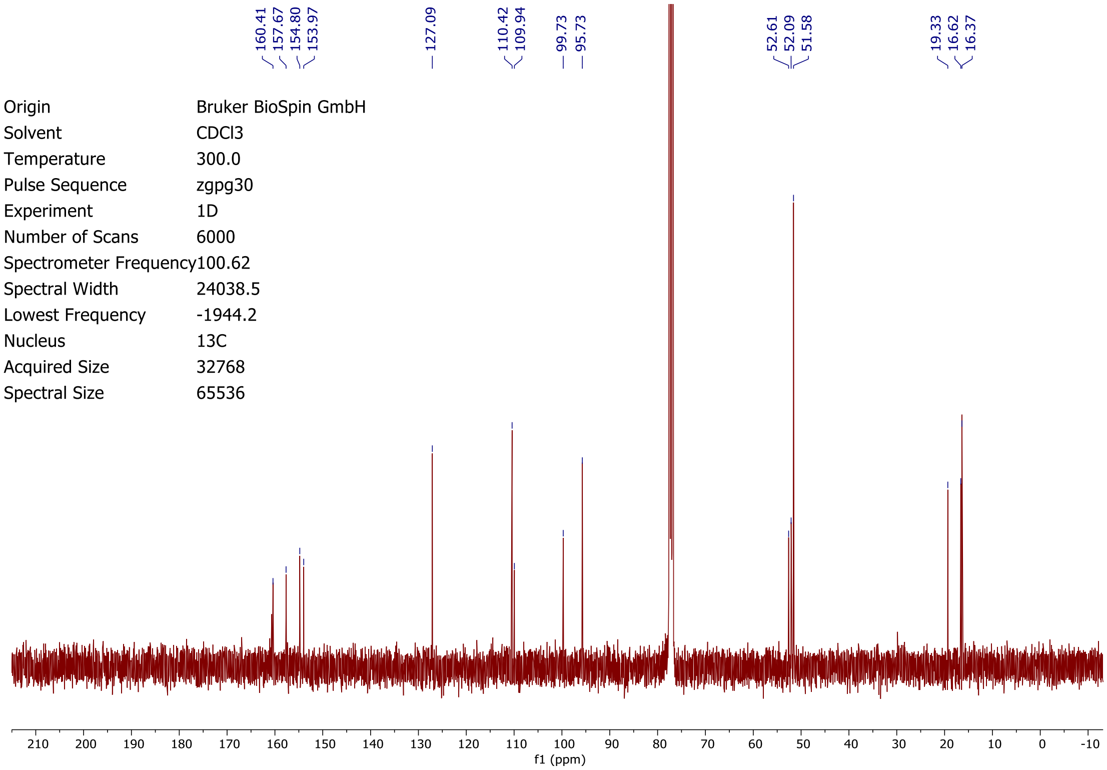

160.41
157.67
154.80
153.97
127.09
110.42
109.94
99.73
95.73
52.61
52.09
51.58
19.33
16.62
16.37
Origin Bruker BioSpin GmbH
Solvent CDCl3
Temperature 300.0
Pulse Sequence zgpg30
Experiment 1D
Number of Scans 6000
Spectrometer Frequency 100.62
Spectral Width 24038.5
Lowest Frequency -1944.2
Nucleus 13C
Acquired Size 32768
Spectral Size 65536
f1 (ppm)

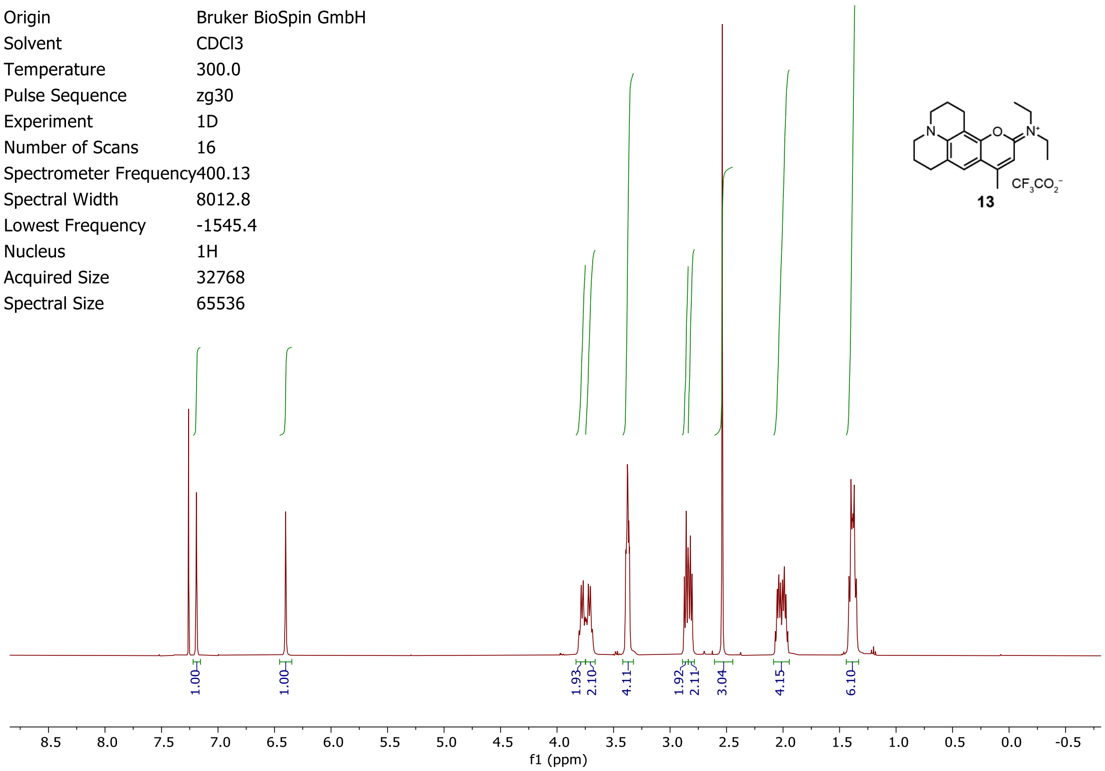
Origin Bruker BioSpin GmbH
Solvent CDCl3
Temperature 300.0
Pulse Sequence zg30
Experiment 1D
Number of Scans 16
Spectrometer Frequency 400.13
Spectral Width 8012.8
Lowest Frequency -1545.4
Nucleus 1H
Acquired Size 32768
Spectral Size 65536
1.00
1.00
1.93
2.10
4.11
1.92
2.11
3.04
4.15
6.10
8.5 8.0 7.5 7.0 6.5 6.0 5.5 5.0 4.5 4.0 3.5 3.0 2.5 2.0 1.5 1.0 0.5 0.0 -0.5
f1 (ppm)
CF3CO2-
13

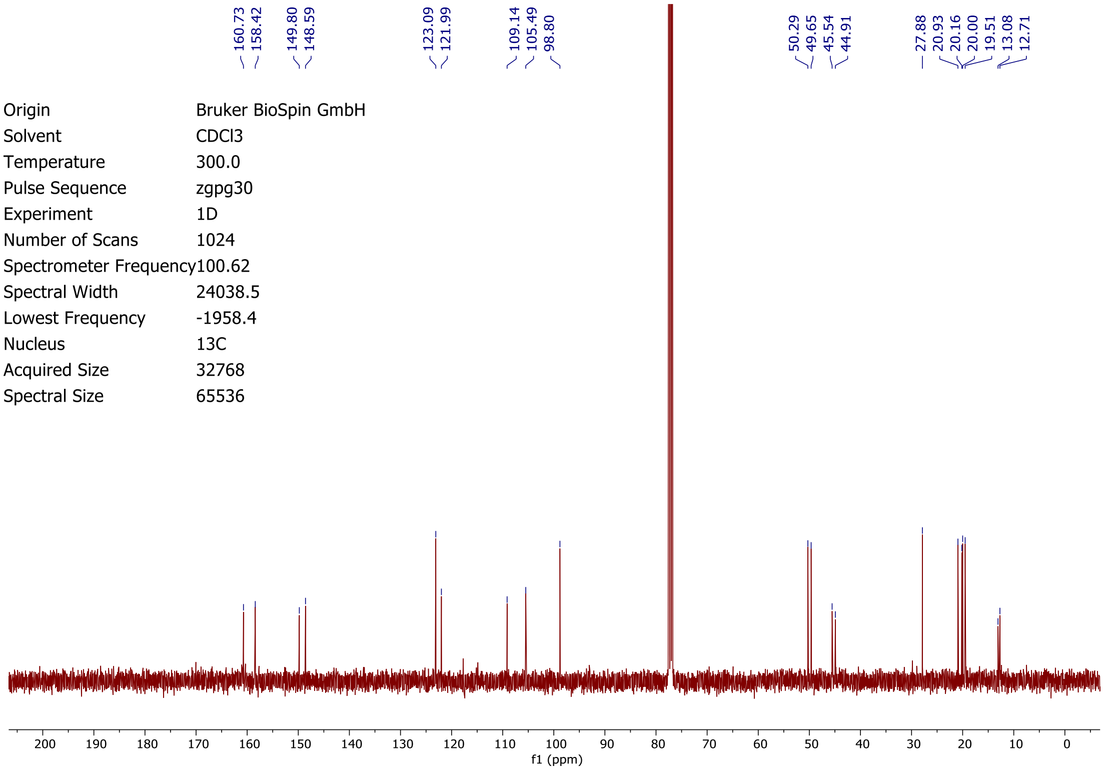
160.73
158.42
149.80
148.59
123.09
121.99
109.14
105.49
98.80
50.29
49.65
45.54
44.91
27.88
20.93
20.16
20.00
19.51
13.08
12.71
Origin Bruker BioSpin GmbH
Solvent CDCl3
Temperature 300.0
Pulse Sequence zgpg30
Experiment 1D
Number of Scans 1024
Spectrometer Frequency 100.62
Spectral Width 24038.5
Lowest Frequency -1958.4
Nucleus 13C
Acquired Size 32768
Spectral Size 65536
200 190 180 170 160 150 140 130 120 110 100 90 80 70 60 50 40 30 20 10 0
f1 (ppm)

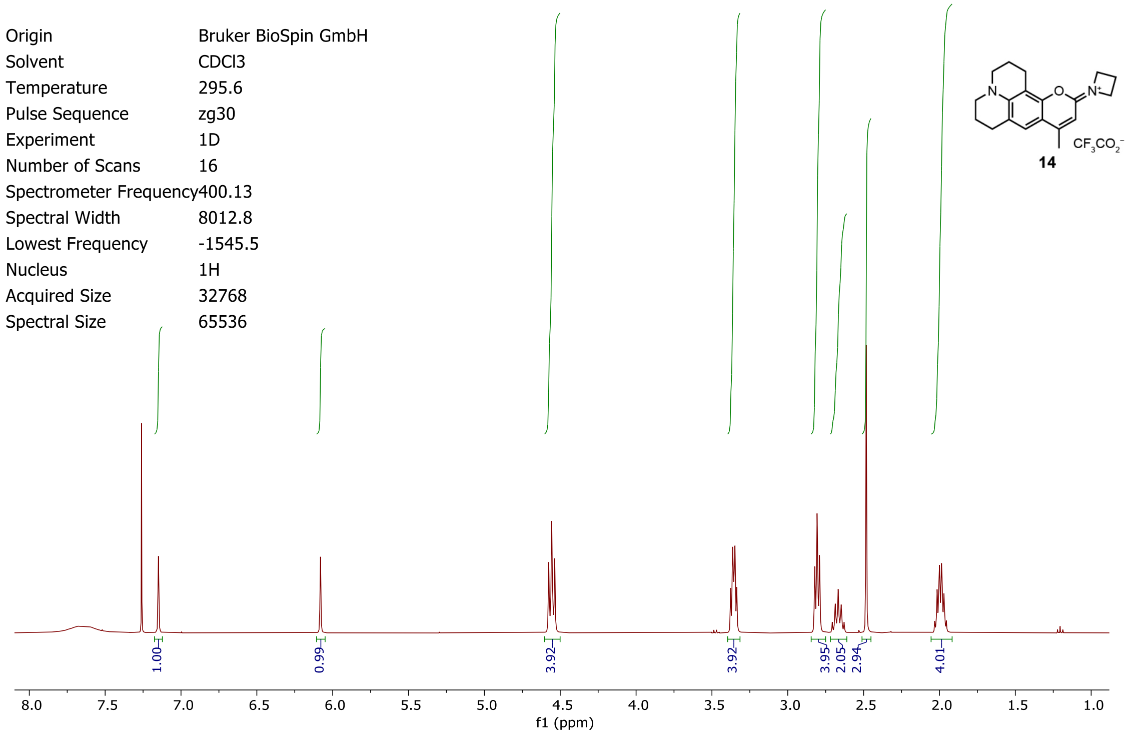
Origin Bruker BioSpin GmbH
Solvent CDCl3
Temperature 295.6
Pulse Sequence zg30
Experiment 1D
Number of Scans 16
Spectrometer Frequency 400.13
Spectral Width 8012.8
Lowest Frequency -1545.5
Nucleus 1H
Acquired Size 32768
Spectral Size 65536
1.00
0.99
3.92
3.92
3.95
2.05
2.94
4.01
8.0 7.5 7.0 6.5 6.0 5.5 5.0 4.5 4.0 3.5 3.0 2.5 2.0 1.5 1.0
f1 (ppm)
N
O
N+
CF3CO2-
14

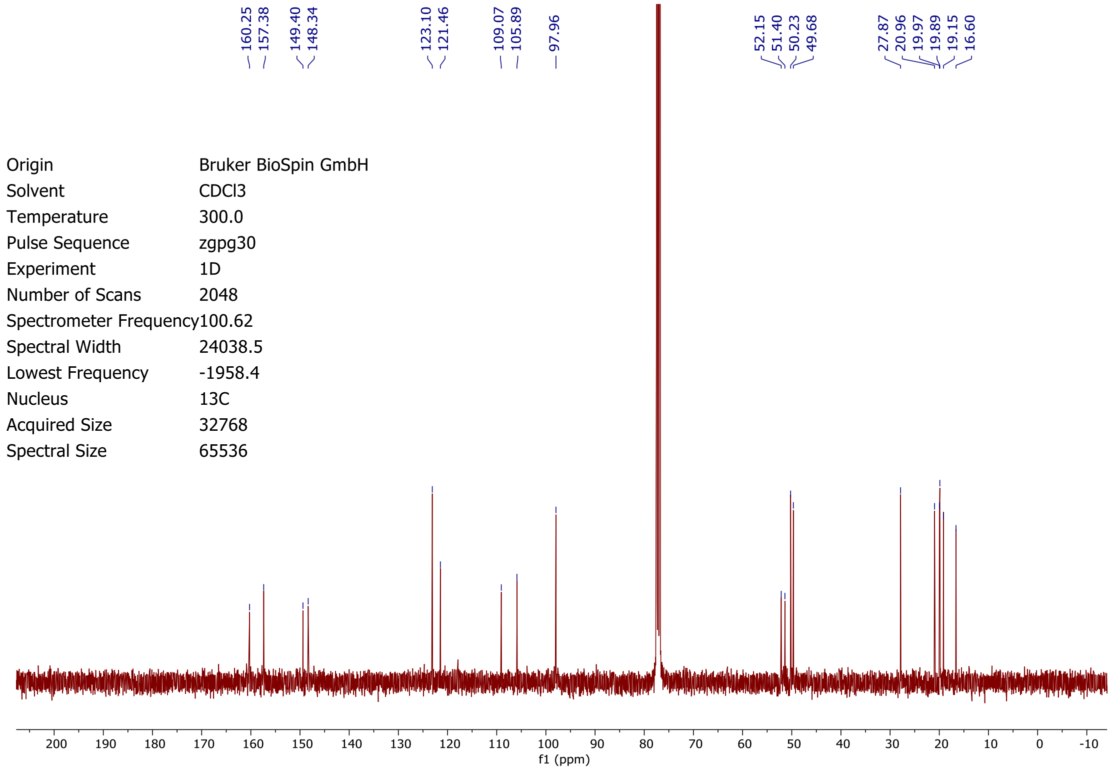
160.25
157.38
149.40
148.34
123.10
121.46
109.07
105.89
97.96
52.15
51.40
50.23
49.68
27.87
20.96
19.97
19.89
19.15
16.60
Origin Bruker BioSpin GmbH
Solvent CDCl3
Temperature 300.0
Pulse Sequence zgpg30
Experiment 1D
Number of Scans 2048
Spectrometer Frequency 100.62
Spectral Width 24038.5
Lowest Frequency -1958.4
Nucleus 13C
Acquired Size 32768
Spectral Size 65536
200 190 180 170 160 150 140 130 120 110 100 90 80 70 60 50 40 30 20 10 0 -10
f1 (ppm)

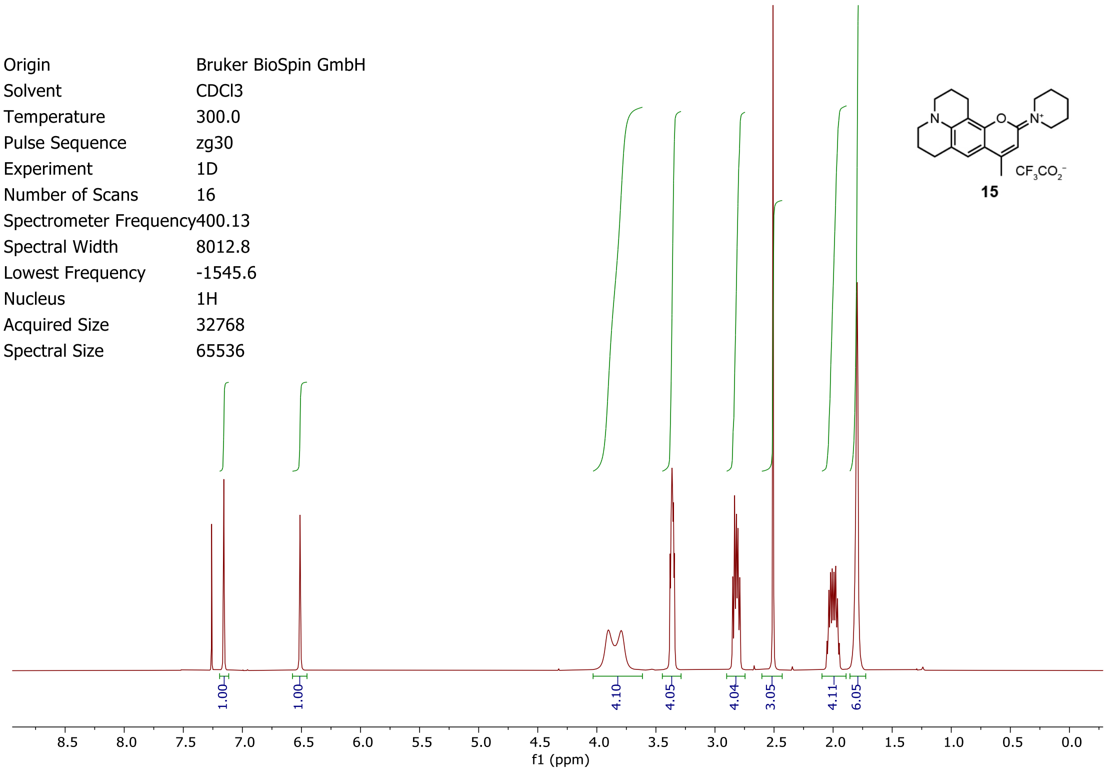
N
O
N+
CF3CO2−
15
Origin Bruker BioSpin GmbH
Solvent CDCl3
Temperature 300.0
Pulse Sequence zg30
Experiment 1D
Number of Scans 16
Spectrometer Frequency 400.13
Spectral Width 8012.8
Lowest Frequency -1545.6
Nucleus 1H
Acquired Size 32768
Spectral Size 65536
1.00
1.00
4.10
4.05
4.04
3.05
4.11
6.05
8.5 8.0 7.5 7.0 6.5 6.0 5.5 5.0 4.5 4.0 3.5 3.0 2.5 2.0 1.5 1.0 0.5 0.0
f1 (ppm)

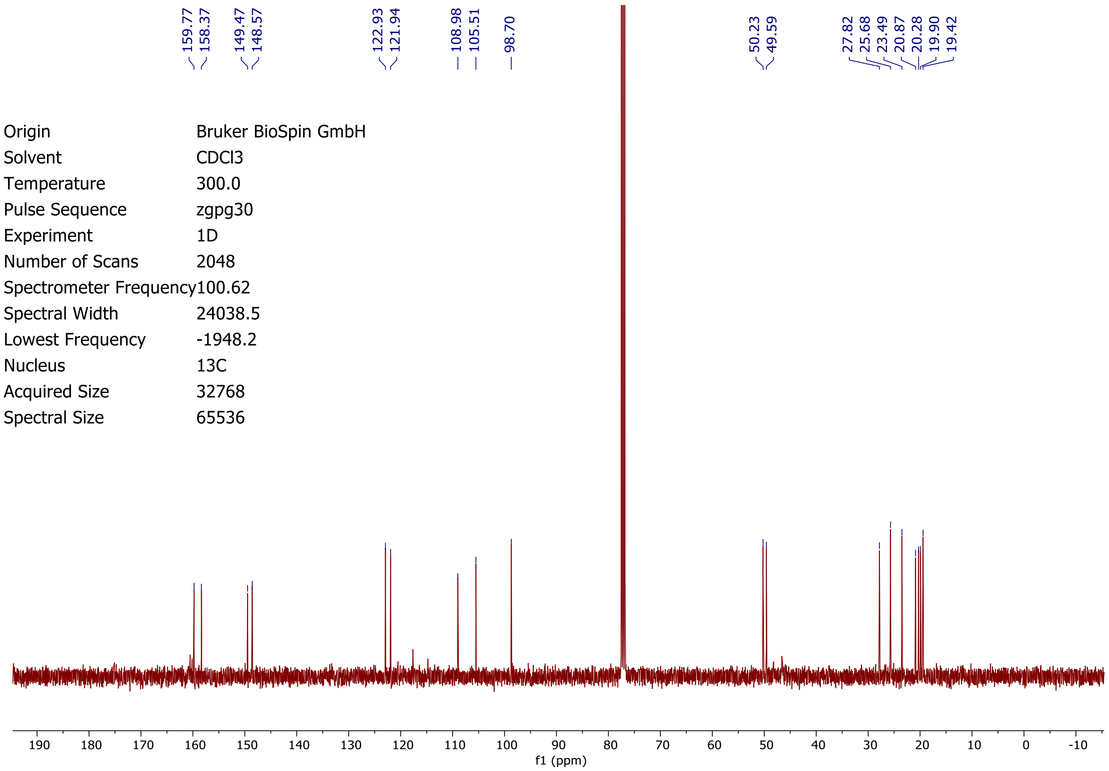
159.77
158.37
149.47
148.57
122.93
121.94
108.98
105.51
98.70
50.23
49.59
27.82
25.68
23.49
20.87
20.28
19.90
19.42
Origin Bruker BioSpin GmbH
Solvent CDCl3
Temperature 300.0
Pulse Sequence zgpg30
Experiment 1D
Number of Scans 2048
Spectrometer Frequency 100.62
Spectral Width 24038.5
Lowest Frequency -1948.2
Nucleus 13C
Acquired Size 32768
Spectral Size 65536
190 180 170 160 150 140 130 120 110 100 90 80 70 60 50 40 30 20 10 0 -10
f1 (ppm)

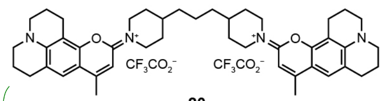

20

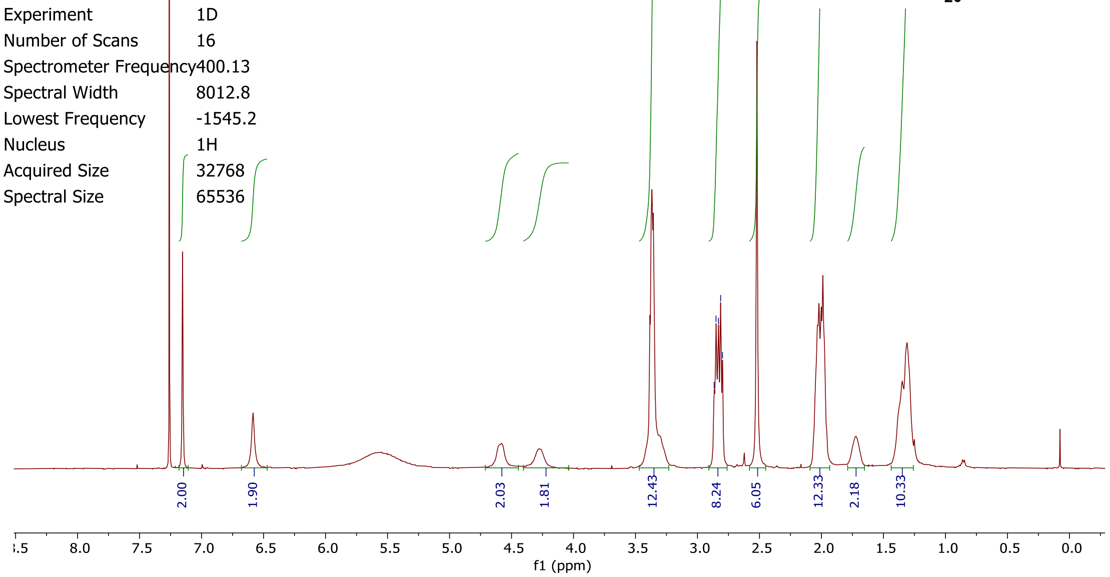

Origin Bruker BioSpin GmbH
Solvent CDCl3
Temperature 300.0
Pulse Sequence zg30
Experiment 1D
Number of Scans 16
Spectrometer Frequency 400.13
Spectral Width 8012.8
Lowest Frequency -1545.2
Nucleus 1H
Acquired Size 32768
Spectral Size 65536
2.00
1.90
2.03
1.81
12.43
8.24
6.05
12.33
2.18
10.33
8.0 7.5 7.0 6.5 6.0 5.5 5.0 4.5 4.0 3.5 3.0 2.5 2.0 1.5 1.0 0.5 0.0
f1 (ppm)

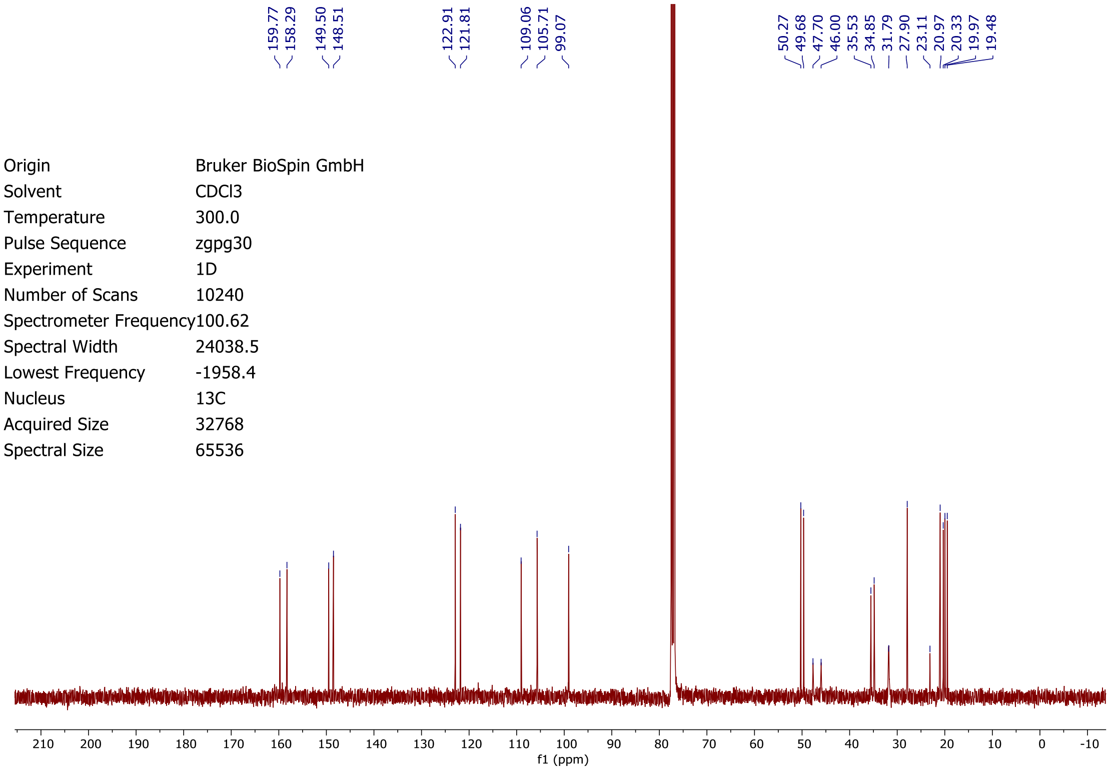

159.77
158.29
149.50
148.51
122.91
121.81
109.06
105.71
99.07
50.27
49.68
47.70
46.00
35.53
34.85
31.79
27.90
23.11
20.97
20.33
19.97
19.48
Origin Bruker BioSpin GmbH
Solvent CDCl3
Temperature 300.0
Pulse Sequence zgpg30
Experiment 1D
Number of Scans 10240
Spectrometer Frequency 100.62
Spectral Width 24038.5
Lowest Frequency -1958.4
Nucleus 13C
Acquired Size 32768
Spectral Size 65536
210 200 190 180 170 160 150 140 130 120 110 100 90 80 70 60 50 40 30 20 10 0 -10
f1 (ppm)
